# Health-related quality of life in the UK Biobank Experience of Pain follow-up study: a comparison with general population norms

**DOI:** 10.1093/aje/kwaf113

**Published:** 2025-05-27

**Authors:** Nigel R Armfield, Scott F Farrell, Belinda J Gabbe, Rachel A Elphinston, Shamini Kosgallana, Luke B Connelly, Michele Sterling

**Affiliations:** RECOVER Injury Research Centre, The University of Queensland, Brisbane, Australia; NHMRC Centre for Excellence in Better Health Outcomes for Compensable Injury, The University of Queensland, Brisbane, Australia; STARS Education and Research Alliance, Surgical Treatment and Rehabilitation Service (STARS), The University of Queensland and Metro North Health, Brisbane, Australia; Centre for Health Services Research, The University of Queensland, Brisbane, Australia; Centre for Innovation in Pain and Health Research (CIPHeR), The University of Queensland, Brisbane, Australia; RECOVER Injury Research Centre, The University of Queensland, Brisbane, Australia; NHMRC Centre for Excellence in Better Health Outcomes for Compensable Injury, The University of Queensland, Brisbane, Australia; STARS Education and Research Alliance, Surgical Treatment and Rehabilitation Service (STARS), The University of Queensland and Metro North Health, Brisbane, Australia; Centre for Innovation in Pain and Health Research (CIPHeR), The University of Queensland, Brisbane, Australia; Tess Cramond Pain and Research Centre, Royal Brisbane and Women’s Hospital, Brisbane, Australia; NHMRC Centre for Excellence in Better Health Outcomes for Compensable Injury, The University of Queensland, Brisbane, Australia; School of Public Health and Preventive Medicine, Monash University, Melbourne, Australia; Population Data Science, Swansea University, Swansea, United Kingdom; RECOVER Injury Research Centre, The University of Queensland, Brisbane, Australia; NHMRC Centre for Excellence in Better Health Outcomes for Compensable Injury, The University of Queensland, Brisbane, Australia; STARS Education and Research Alliance, Surgical Treatment and Rehabilitation Service (STARS), The University of Queensland and Metro North Health, Brisbane, Australia; Centre for Innovation in Pain and Health Research (CIPHeR), The University of Queensland, Brisbane, Australia; RECOVER Injury Research Centre, The University of Queensland, Brisbane, Australia; NHMRC Centre for Excellence in Better Health Outcomes for Compensable Injury, The University of Queensland, Brisbane, Australia; STARS Education and Research Alliance, Surgical Treatment and Rehabilitation Service (STARS), The University of Queensland and Metro North Health, Brisbane, Australia; Centre for Innovation in Pain and Health Research (CIPHeR), The University of Queensland, Brisbane, Australia; NHMRC Centre for Excellence in Better Health Outcomes for Compensable Injury, The University of Queensland, Brisbane, Australia; Centre for Innovation in Pain and Health Research (CIPHeR), The University of Queensland, Brisbane, Australia; Centre for the Business and Economics of Health, The University of Queensland, Brisbane, Australia; School of Sociology and Business Law, University of Bologna, Bologna, Italy; RECOVER Injury Research Centre, The University of Queensland, Brisbane, Australia; NHMRC Centre for Excellence in Better Health Outcomes for Compensable Injury, The University of Queensland, Brisbane, Australia; Centre for Health Services Research, The University of Queensland, Brisbane, Australia; Centre for Innovation in Pain and Health Research (CIPHeR), The University of Queensland, Brisbane, Australia

**Keywords:** HRQoL, UK Biobank, EQ-5D, representativeness, participation bias, healthy volunteer effect

## Abstract

The UK Biobank (UKB) is a population-based resource of genetic, health, lifestyle, sociodemographic, and linked clinical data for approximately 500 000 volunteers. Previous analyses of health determinants have shown that the UKB is not representative of the population from which it is drawn. However, representativeness from a general health-outcomes perspective is unknown, but could be assessed using health-related quality of life (HRQoL) questionnaire responses and expected population norms. The HRQoL responses, collected using the EuroQoL EQ-5D-5L instrument, were available for participants who completed the *experience of pain* follow-up survey (*n* = 167 199). We comprehensively characterized follow-up participant HRQoL and made age and sex-specific comparisons with normative data derived from the nationally representative Health Survey for England (2014). The pattern of reported problems mirrored those of the expected population norms, but with differences in magnitude: for both sexes, at all ages (except >74 years) the prevalence/odds of problems were greater for UKB participants. Compared with the norms, HRQoL of participants in this subsample did not follow a pattern of decline as expected with increasing age. While we did find differences in patterns between the EoPQ cohort and expected population HRQoL, the absolute differences were only modest, and this should provide reassurance for users of the data.

## Introduction

The UK Biobank (UKB) is a population-based prospective cohort of approximately 500 000 volunteers in England, Scotland, and Wales with participant-level genetic, health, lifestyle, sociodemographic, and linked clinical data. Recruitment was by postal invitation to 9.2 million adults aged 40-69 years identified from National Health Service (NHS) records with baseline assessments conducted during 2006-2010.^1^^,^^2^ The UKB has over 38 000 registered researchers (16% UK; 84% international); over 10 000 scientific papers report using its data.^1^

The UKB design strengths include the recruitment of participants at a young enough age such that few would yet have developed major health conditions, and a long study duration allowing a large number of cases of common conditions to occur during follow-up. Further, because of the breadth and depth of the data, a wide range of risk factors can be assessed before diseases develop, the full effects of exposures on health outcomes can be assessed, as can the effects of many different exposures on a single disease. Finally, a wide range of conditions may be studied in the same study population.^1^

While the UKB is well designed, the participation rate is low (~5.5% completed baseline assessments).^1^^,^^2^ Previous studies have identified selection bias, particularly healthy volunteer bias, whereby participants have characteristics favoring better health than those of the wider population from which they are drawn.^3^^-^^7^

Two studies have explored the population-representativeness of the UKB in detail^5^^,^^8^: Fry *et al*.^5^ compared the sociodemographic and health-related characteristics of UKB respondents with nonrespondents, finding that respondents were more likely to be female, older, and live in less socioeconomically deprived areas. When comparing with the nationally representative *Health Survey for England* (HSE),^9^ Fry *et al*. also found that lifestyle-related risk factors of UKB respondents differed to the general population; respondents were less likely to be living with obesity, smoke, drink alcohol daily, and had fewer self-reported health issues.^5^ Davis *et. al*^8^ found that respondents to the UKB mental health-focused *Thoughts and Feelings* follow-up questionnaire (2016)^10^ had a higher level of education, higher socioeconomic status, were healthier, and had lower rates of smoking compared to the UKB as a whole, and compared to the UK general population.^8^

These prior comparisons were made using *determinants* of health, that is, the influences on the health that the cohort may possibly experience sometime in the future. However, it remains unclear whether, or how, the *actual experienced* health of participants compares with the general population. It is possible to make this comparison using responses to health-related quality of life (HRQoL) questionnaires and normative population data; doing so would add important new knowledge about the UKB’s population representativeness. Additionally, HRQoL responses have another very important role: by comparison with normative data they can be used to quantify the loss of good health (ie, disease burden) associated with specific health conditions, and conclusions may be generalized and used to inform policy and practice.^11^

Within the UKB, HRQoL data were not collected at baseline and hence it is not possible to make a comparison with population norms for the entire cohort. However, the EuroQoL EQ-5D (specifically, EQ-5D-5L), a generic multiattribute utility instrument for measuring HRQoL^12^^-^^14^ was included in the UKB *Experience of pain* follow-up questionnaire (EoPQ; completed by 167 199 participants).^15^ Within the EoPQ cohort, future studies could quantify the loss of good health associated with chronic pain conditions, identify predictive factors associated with those losses of good health, and answer other important questions related to the burden of chronic pain. It is important that conclusions of studies are valid and reliable.

To our knowledge, the HRQoL of UKB participants has not been quantified and described, and it is unknown if, and how, the cohort HRQoL departs from expected population norms. Our motivation is to address this important knowledge gap and to provide new comprehensive reference information of relevance to users of the EoPQ data, and potentially other UKB follow-up studies. Our aim was to describe the HRQoL of EoPQ respondents, and to compare it with expected age and sex-standardized population norms derived from the HSE.

## Methods

### Data sources

#### UK Biobank

Baseline data were collected using self-response touchscreen questionnaires, study nurse interviews, and anthropometric measurements. Biospecimens (blood, urine, saliva) were also collected. Follow-up data are collected through repeat assessments, imaging, physical activity monitoring, online questionnaires, and by data linkage to health and other registries and records.^16^ Participants resided within 25 miles (40 km) of one of 22 assessment centers; 91% of assessments were conducted in England (6% Scotland, 3% Wales).^17^

In 2019*,* approximately 330 000 participants were invited to complete the EoPQ,^15^ which focused primarily on the experience of chronic pain and incorporated instruments validated in pain cohorts; respondents included both those with and without experience of chronic pain.

We extracted baseline characteristics for all UKB participants, comprising sociodemographic, environmental, and lifestyle risk factors and health determinants (age, sex, ethnic background, education, employment status, pretax household income, Townsend Deprivation Index (an area-based measure of socioeconomic deprivation),^18^ Body Mass Index (BMI), smoking status, alcohol consumption frequency, and physical activity (International Physical Activity Questionnaire, IPAQ).^19^ We also extracted linked hospital-admission diagnosis codes (International Classification of Diseases 9th/10th Revisions, ICD-9, ICD-10 with diagnosis date ≤baseline assessment date^20^^,^^21^ to calculate comorbidity scores (Charlson Comorbidity Index, CCI),^22^ reported in categories (0, *none*; 1-2; 3-4; ≥5).^23^ Finally, we extracted the HRQoL questionnaire (EQ-5D-5L) responses for the EoPQ respondent subsample.^15^ Participants who withdrew from the UKB prior to our analysis were excluded per UKB requirements. There was no censoring for death.

### Overview of EuroQoL EQ-5D

Here we provide a brief overview of the EuroQoL EQ-5D instruments; an in-depth description and glossary of terms is provided in Appendix S1. The EQ-5D has 2 components, the *descriptive system* (described below) and the *EQ-VAS* (not used in this study).

Both the EQ-5D-3L (used in the HSE) and EQ-5D-5L (used in the UKB) ask respondents to report their “health today” along 5 dimensions (*mobility, self-care, usual activities, pain/discomfort, anxiety/depression).* For each dimension, the EQ-5D-3L allows for 3 levels of response (*no problems, some problems, unable/extreme*); the EQ-5D-5L has 5 levels of response (*none*, *slight*, *moderate, severe*, *unable/extreme)*. Scoring and summarization is standardized and described in the EQ-5D user guides^24^; briefly, for EQ-5D-3L, responses are scored using a value between 1 and 3 (EQ-5D-5L, 1 and 5) with “1” representing “no problems”. For each respondent, “health today” is summarized using a 5-digit *health state* (also known as *EQ-5D health profile*) by concatenating the item responses (eg, EQ-5D-3L response with some *mobility* problems (score 2), no *self-care* problems (1), some problems with *usual activities* (2) and *pain/discomfort* (2), no problems with anxiety/depression (1) would be represented by EQ-5D-3L *health state* “21221”). Example scoring is shown in Table S1 and Figure S1.

#### Health utilities

A health state may be translated into a population-specific summary value, known as a *health utility*. Health utilities (also known as *EQ-5D value,* or *health state value*) represent a value or preference that an individual or society gives to being in a particular *health state*^25^ with “1” representing *full health*, “0” being *as bad as being dead*, and values <0 representing a state *worse than being dead*. Utilities are computed using weights from population-specific *value sets*, also known as *tariffs*. Health utilities are commonly used to summarize population health. While the economics literature uses the term “utility” to describe health-related preferences that incorporate uncertainty,^26^ the general literature uses the terms such as *health utility* and *health state value* interchangeably; here we have chosen to use *health utility* to be consistent with common usage within the EQ-5D literature that we cite.

#### Normative data

Normative EQ-5D-5L utilities for England (most UKB participants resided in England) have recently been derived from the HSE 2017/2018,^27^ but to our knowledge, expected normative values for the EQ-5D-5L *descriptive system* for England have not been published. We used the most recent publicly available HSE with EQ-5D responses (HSE2014; EQ-5D-3L) to derive comprehensive population norms. The HSE2014 was a stratified random probability sample of private households, but not institutions (9024 addresses in 564 postcode sectors, 62% household response rate of 8077 adults and 2003 children). The survey contained weights to allow correction for nonresponse bias, calibrated to the population estimates for age, sex, and region using the 2013 Office of National Statistics midyear population estimates.^28^ Although the UKB and HSE2014 used different versions of the EQ-5D instruments, mapping algorithms allow comparisons to be made.

Data source references are tabulated in Appendix S2 (Table S2).

### Analysis

#### Participant characteristics

We tabulated participant characteristics (baseline assessment) for the complete UKB sample, and then separately for the EoPQ subsample and the non-EoPQ subsample (participants who were either not invited to complete the EoPQ, or who were invited but did not respond). In our dataset, we were unable to identify the subset of participants who were invited, but who did not respond, or the subset without an email address who were unable to be invited. We compared the characteristics of the EoPQ and non-EoPQ subsamples (independent 2-sample *t*-tests for continuous variables, chi-squared for categorical variables).

#### EQ-5D

We grouped participants by age (45-54, 55-64, 65-74, and >74 years) and tabulated raw frequencies and proportions by EQ-5D descriptive dimension by level of problem, age group, and sex. We then dichotomised the level of problem (*none* to *no problems* and *slight*, *moderate, severe*, *unable/extreme* to *some problems*), retabulated frequencies and proportions, and used logistic regression to estimate the odds/95% CIs of reporting problems by increasing age for each dimension separately by sex (references: *ages 45-54 years*, *no problems*).

A value set for England published by Devlin *et al*. allows direct valuation (ie, derivation of utilities) of EQ-5D-5L health states,^29^ however it is not supported by the National Institute for Health and Care Excellence (NICE).^30^^,^^31^ Two alternative crosswalk-based approaches exist (van Hout *et al*.^32^ and Hernandez Alava *et al*.,^33^ currently preferred by NICE). Both methods map EQ-5D-5L health states to the Dolan 1997 EQ-5D-3L value set for the United Kingdom^34^.

**Figure 1:**
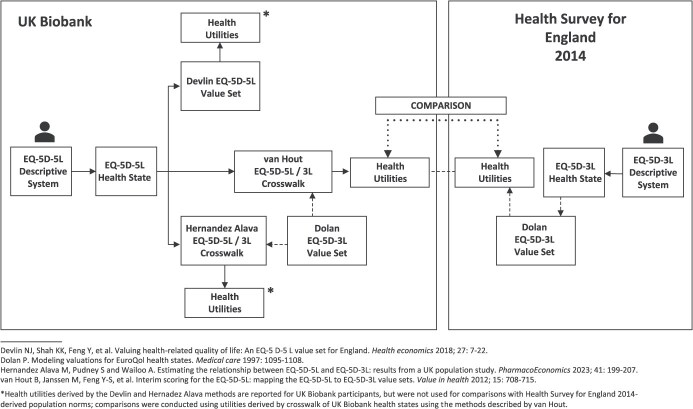
Flowchart of EQ-5D calculations, crosswalks, and comparisons between the UK Biobank and expected population norms.

To accommodate all preferences, we adopted a method-neutral approach, and reported utilities using all 3 (Devlin, van Hout, Hernandez Alava) methods. For comparison with population norms we used only the van Hout-derived utilities^32^ (process shown in Figure 1).

#### Derivation of expected population EQ-5D norms

Descriptive system norms were estimated for Adult HSE2014 respondents with ≥1 EQ-5D descriptive response; utility norms were computed for those with complete responses. We again dichotomised level of problem and tabulated raw counts, and weighted proportions using HSE2014 individual participant weights.^28^ To allow direct comparison with the UKB, we then excluded respondents aged <45 years and again used logistic regression to estimate the odds/95% CIs of reporting problems by increasing age for each dimension by sex.

We computed utilities (Dolan 1997 UK value set)^34^ for adult HSE respondents and then estimated expected population utilities separately for females and males (ages ≥18-90 years) by fitting quadratic regression models with age and age-squared as explanatory variables to model the nonlinear effect of age. Finally, we then estimated expected population utilities by age group (45-54, 55-64, 65-74, and 74 years) using group midpoints derived from the regression models.

### Comparison of UKB HRQoL with expected population norms

#### Descriptive system

For each dimension, by age group, and sex, we used radar plots to present the prevalence of reported problems (UKB vs normative equivalents, dichotomised; *no problems*, *some problems*). We then estimated and tabulated ORs/95% CIs of reporting problems by sex and age group, with normative values as the references.

#### Health utilities

For UKB participants, we used the van Hout *et al*. cross-walked health utilities described above.^32^ For each participant, we subtracted their utility from the corresponding age and sex-specific expected population utility previously estimated by quadratic regression. Results were plotted using histograms by sex. Depending on distributional assumptions, we used either a 1-sample *t*-test or the nonparametric equivalent 1-sample Wilcoxon test to assess whether any UKB deviations from the expected population norms differed significantly from zero. Finally, we calculated mean health utilities by age group and sex and plotted them against their corresponding group midpoint expected population norms.

All data were and analyzed using R version 4.3.2.^23^^,^^35^^-^^38^

## Results

### Participant characteristics

#### UK Biobank

The complete UKB sample comprised 502 411 participants (EoPQ subsample, *n* = 167 199; females *n* = 94 998 [56.82%], males *n* = 72 201 [43.18%]; response rate of ~51%; Non-EPoQ subsample, *n* = 335 212 participants). Table 1 shows participant characteristics (complete sample, baseline assessment).

**Table 1:** Participant characteristics at baseline assessment, complete UK Biobank sample.

	Complete UK Biobank sample (*n* = 502 411)					
	Females		Males		Total	
Participants, *n* %	273 325	54.40	229 086	45.60	502 411	100.00
Age (y), mean, SD	56.30	8	56.70	8.20	56.50	8.1
*Missing, n %*	*1*	*<0.1*	*2*	*<0.1*	*3*	*<0.1*
Ethnic background, *n* %						
White (British, Irish, other)	257 388	94.17	215 224	93.95	472 612	94.07
Mixed	1850	0.68	1104	0.48	2954	0.59
Asian or Asian British	4585	1.68	5294	2.31	9879	1.97
Black or Black British	4652	1.70	3406	1.49	8058	1.60
Chinese	989	0.36	584	0.25	1573	0.31
Other ethnic group	2595	0.95	1962	0.86	4557	0.91
Do not know	107	0.04	110	0.05	217	0.04
Prefer not to answer	706	0.26	955	0.42	1661	0.33
*Missing*	*453*	*0.17*	*447*	*0.20*	*900*	*0.18*
Highest level of education, *n* %						
Degree	84 512	30.92	76 616	33.44	161 128	32.07
A/AS Level	31 996	11.71	23 312	10.18	55 308	11.01
O/GCSE	63 110	23.09	42 066	18.36	105 176	20.93
CSE	14 597	5.34	12 288	5.36	26 885	5.35
NVQ/HNC/HND	12 167	4.45	20 557	8.97	32 724	6.51
Other professional	15 643	5.72	10 156	4.43	25 799	5.14
None of these	45 959	16.81	39 300	17.16	85 259	16.97
Prefer not to answer	2849	1.04	2641	1.15	5490	1.09
*Missing*	*2492*	*0.91*	*2150*	*0.94*	*4642*	*0.92*
Current employment status, *n* %						
Paid employment/self-employed	149 159	54.57	137 923	60.21	287 082	57.14
Retired	95 552	34.96	71 416	31.17	166 968	33.23
Looking after home/or family	12 622	4.62	1233	0.54	13 855	2.76
Unable (sickness/disability)	7452	2.73	9371	4.09	16 823	3.35
Unemployed	2889	1.06	5374	2.35	8263	1.64
Unpaid or voluntary work	1670	0.61	656	0.29	2326	0.46
Full or part-time student	882	0.32	461	0.20	1343	0.27
None of the above	1581	0.58	1219	0.53	2800	0.56
Prefer not to answer	1078	0.39	1001	0.44	2079	0.41
*Missing*	*440*	*0.16*	*432*	*0.19*	*872*	*0.17*
Income (GBP)^a^, *n*%						
> 100 000	10 720	3.92	12 207	5.33	22 927	4.56
52 000-100 000	41 618	15.23	44 632	19.48	86 250	17.17
31 000-51 999	56 751	20.76	54 002	23.57	110 753	22.04
18 000-30 999	58 814	21.52	49 343	21.54	108 157	21.53
< 18 000	55 404	20.27	41 776	18.24	97 180	19.34
Do not know	15 645	5.72	5655	2.47	21 300	4.24
Prefer not to answer	31 228	11.43	18 602	8.12	49 830	9.92
*Missing*	*3145*	*1.15*	*2869*	*1.25*	*6014*	*1.20*
Townsend deprivation index, *n* %						
<–2 (least deprived)	141 887	51.91	118 177	51.59	260 064	51.76
–2 to <2	87 785	32.12	71 627	31.27	159 412	31.73
≥2	43 325	15.85	38 984	17.02	82 309	16.38
*Missing*	*328*	*0.12*	*298*	*0.13*	*626*	*0.12*
Charlson comorbidity index, *n* %						
0	250 500	91.65	203 000	88.61	453 500	90.26
1-2	21 335	7.81	23 195	10.13	44 530	8.86
3-4	1241	0.45	2238	0.98	3479	0.69
≥5	249	0.09	653	0.29	902	0.18
BMI (kg/m^2^), *n* %						
<18.5	2079	0.76	547	0.24	2626	0.52
≥18.5 and <25	105 638	38.65	56 733	24.76	162 371	32.32
≥25 and <30	99 852	36.53	112 226	48.99	212 078	42.21
≥30	64 297	23.52	57 932	25.29	122 229	24.33
*Missing*	*1459*	*0.53*	*1648*	*0.72*	*3107*	*0.62*
Participants, *n* %	273 325	54.40	229 086	45.60	502 411	100.00
Smoking status, *n* %						
Never	162 022	59.28	111 453	48.65	273 475	54.43

**Table 1:** Continued

	Complete UK Biobank sample (*n* = 502 411)					
	Females		Males		Total	
Previous	85 429	31.26	87 595	38.24	173 024	34.44
Current	24 361	8.91	28 601	12.48	52 962	10.54
Prefer not to answer	1062	0.39	995	0.43	2057	0.41
*Missing*	*451*	*0.17*	*442*	*0.19*	*893*	*0.18*
Alcohol frequency, *n* %						
Daily/almost daily	43 856	16.05	57 897	25.27	101 753	20.25
3-4 times/week	55 885	20.45	59 537	25.99	115 422	22.97
1-2 times/week	70 164	25.67	59 107	25.80	129 271	25.73
1-3 times/month	35 494	12.99	20 346	8.88	55 840	11.11
Special occasions only	41 144	15.05	16 852	7.36	57 996	11.54
Never	26 043	9.53	14 584	6.37	40 627	8.09
Prefer not to answer	286	0.10	317	0.14	603	0.12
*Missing*	*453*	*0.17*	*446*	*0.19*	*899*	*0.18*
Physical activity (IPAQ), *n* %						
Low	39 372	14.40	36 821	16.07	76 193	15.17
Moderate	90 506	33.11	73 493	32.08	163 999	32.64
High	80 875	29.59	81 236	35.46	162 111	32.27
*Missing*	*62 572*	*23.89*	*37 536*	*16.39*	*100 108*	*19.93*

A/AS Level, Advanced/Advanced Subsidiary Level; BMI, body mass index; CSE, Certificate of Secondary Education; HNC, Higher National Certificate; HND, Higher National Diploma; IPAQ, International Physical Activity Questionnaire; NVQ, National Vocational Qualification; O/GCSE, Ordinary/General Certificate of Education.

a Pretax household income; GBP, British pounds sterling.

The EoPQ and the non-EoPQ subsamples differed in important ways; except for alcohol consumption, the non-EoPQ subsample had a greater proportion of participants with risk factors and social determinants known to be associated with poorer health, and hence diminished quality of life (Table 2).

**Table 2:** Participant characteristics at baseline assessment, UK Biobank EoPQ and non-EoPQ subsamples.

	UK Biobank EoPQ follow-up subsample (*n* = 167 199)						UK Biobank non-EoPQ follow-up subsample (*n* = 335 212)						*P*	
	Females		Males		Total		Females		Males		Total		Females	Males
Participants, *n* %	94 998	56.82	72 201	43.18	167 199	100.00	178 327	53.20	156 885	46.80	335 212	100.00		
Age (y), mean, SD	55.32	7.62	56.27	7.75	55.73	7.69	56.89	8.15	56.96	8.39	56.93	8.26	<.001	<.001
*Missing, n %*	*0*	*0*	*0*	*0*	*0*	*0*	*1*	*<0.1*	*2*	*<0.1*	*3*	*<0.1*		
Ethnic background, *n* %														
White (British, Irish, other)	91 950	96.79	69 783	96.65	161 733	96.73	165 498	92.81	145 411	92.69	310 909	92.75	*χ^2^* = 1941 *df* = 7 *P* <.001	*χ^2^* = 1385 *df* = 7 *P* <.001
Mixed	567	0.60	288	0.40	855	0.51	1283	0.72	816	0.52	2099	0.63		
Asian or Asian British	670	0.71	805	1.11	1475	0.88	3853	2.16	4489	2.86	8342	2.49		
Black or Black British	734	0.77	492	0.68	1226	0.73	3918	2.20	2914	1.86	6832	2.04		
Chinese	267	0.28	120	0.17	387	0.23	722	0.40	464	0.30	1186	0.35		
Other ethnic group	552	0.58	374	0.52	926	0.55	2043	1.15	1588	1.01	3631	1.08		
Do not know	21	0.02	30	0.04	51	0.03	86	0.05	80	0.05	166	0.05		
Prefer not to answer	192	0.20	278	0.39	470	0.28	514	0.29	677	0.43	1191	0.36		
*Missing*	*45*	*0.05*	*31*	*0.04*	*76*	*0.05*	*410*	*0.23*	*446*	*0.28*	*856*	*0.26*		
Highest level of education, *n* %														
Degree	41 331	43.51	34 059	47.17	75 390	45.09	43 181	24.21	42 557	27.13	85 738	25.58	*χ^2^* = 18 942 *df* = 7 *P* <.001	*χ^2^* = 13 787 *df* = 7 *P* <.001
A/AS Level	13 527	14.24	8677	12.02	22 204	13.28	18 469	10.36	14 635	9.33	33 104	9.88		
O/GCSE	20 616	21.70	12 402	17.18	33 018	19.75	42 494	23.83	29 664	18.91	72 158	21.53		
CSE	3646	3.84	2739	3.79	6385	3.82	10 951	6.14	9549	6.09	20 500	6.12		
NVQ/HNC/HND	2987	3.14	5581	7.73	8568	5.12	9180	5.15	14 976	9.55	24 156	7.21		
Other professional	5471	5.76	2868	3.97	8339	4.99	10 172	5.70	7288	4.65	17 460	5.21		
None of these	6488	6.83	5170	7.16	11 658	6.97	39 471	22.13	34 130	21.75	73 601	21.96		
Prefer not to answer	283	0.30	256	0.35	539	0.32	2566	1.44	2385	1.52	4951	1.48		
*Missing*	*649*	*0.68*	*449*	*0.62*	*1098*	*0.66*	*1843*	*1.03*	*1701*	*1.08*	*3544*	*1.06*		
Current employment status, *n* %														
Working/self-employed	59 939	63.10	47 901	66.34	107 840	64.50	89 220	50.03	90 022	57.38	179 242	53.47	*χ^2^* = 4960 *df* = 8 *P* <.001	*χ^2^* = 2854 *df* = 8 *P* <.001
Retired	26 940	28.36	20 779	28.78	47 719	28.54	68 612	38.48	50 637	32.28	119 249	35.57		
Looking after home/or family	4270	4.49	318	0.44	4588	2.74	8352	4.68	915	0.58	9267	2.76		
Unable (sickness/disability)	1389	1.46	1153	1.60	2542	1.52	6063	3.40	8218	5.24	14 281	4.26		
Unemployed	748	0.79	1207	1.67	1955	1.17	2141	1.20	4167	2.66	6308	1.88		
Unpaid or voluntary work	706	0.74	225	0.31	931	0.56	964	0.54	431	0.27	1395	0.42		
Full- or part-time student	336	0.35	120	0.17	456	0.27	546	0.31	341	0.22	887	0.26		
None of the above	464	0.49	339	0.47	803	0.48	1117	0.63	880	0.56	1997	0.60		
Prefer not to answer	162	0.17	129	0.18	291	0.17	916	0.51	872	0.56	1788	0.53		
*Missing*	*44*	*0.05*	*30*	*0.04*	*74*	*0.04*	*396*	*0.22*	*402*	*0.26*	*798*	*0.24*		
Participants, *n* %	94 998	56.82	72 201	43.18	167 199	100.00	178 327	53.20	156 885	46.80	335 212	100.00		
Income (GBP)^a^, *n* %														
> 100 000	5843	6.15	6043	8.37	11 886	7.11	4877	2.73	6164	3.93	11 041	3.29	*χ^2^* = 12 722 *df* = 6 *P* <.001	*χ^2^* = 10 875 *df* = 6 *P* <.001
52 000-100 000	20 322	21.39	19 286	26.71	39 608	23.69	21 296	11.94	25 346	16.16	46 642	13.91		
31 000-51 999	23 825	25.08	19 894	27.55	43 719	26.15	32 926	18.46	34 108	21.74	67 034	20.00		
18 000-30 999	20 346	21.42	14 295	19.80	34 641	20.72	38 468	21.57	35 048	22.34	73 516	21.93		
<18 000	12 981	13.66	7595	10.52	20 576	12.31	42 423	23.79	34 181	21.79	76 604	22.85		
Do not know	3065	3.23	672	0.93	3737	2.24	12 580	7.05	4983	3.18	17 563	5.24		
Prefer not to answer	7890	8.31	3916	5.42	11 806	7.06	23 338	13.09	14 686	9.36	38 024	11.34		
*Missing*	*726*	*0.76*	*500*	*0.69*	*1226*	*0.73*	*2419*	*1.36*	*2369*	*1.51*	*4788*	*1.43*		

**Table 2:** Continued

	UK Biobank EoPQ follow-up subsample (*n* = 167 199)						UK Biobank non-EoPQ follow-up subsample (*n* = 335 212)						*P*	
	Females		Males		Total		Females		Males		Total		Females	Males
Townsend deprivation index, *n* %														
<–2 (least deprived)	52 721	55.5	41 393	57.33	94 114	56.29	89 166	50.00	76 784	48.94	165 950	49.51	*χ^2^* = 1401 *df* = 3 *P* <.001	*χ^2^* = 2099 *df* = 3 *P* <.001
–2 to <2	30 303	31.90	21 860	30.28	52 163	31.20	57 482	32.23	49 767	31.72	107 249	31.99		
≥2	11 866	12.49	8869	12.28	20 735	12.40	31 459	17.64	30 115	19.20	61 574	18.37		
*Missing*	*108*	*0.11*	*79*	*0.11*	*187*	*0.11*	*220*	*0.12*	*219*	*0.14*	*439*	*0.13*		
Charlson comorbidity index, *n* %														
0	89 480	94.19	66 796	92.51	156 276	93.47	161 020	90.29	136 204	86.82	297 224	88.67	*χ^2^* = 1284 *df* = 3 *P* <.001	*χ^2^* = 1665 *df* = 3 *P* <.001
1-2	5280	5.56	5010	6.94	10 290	6.15	16 053	9.0	18 185	11.59	34 238	10.21		
3-4	213	0.22	298	0.41	511	0.31	1028	0.58	1940	1.24	2968	0.89		
≥5	23	0.02	97	0.13	120	0.07	226	0.13	556	0.35	782	0.23		
BMI (kg/m^2^), *n* %														
<18.5	809	0.85	128	0.18	937	0.56	1270	0.71	419	0.27	1689	0.50	*χ^2^* = 3133 *df* = 4 *P* <.001	*χ^2^* = 2001 *df* = 4 *P* <.001
≥18.5 and <25	42 705	44.95	21 162	29.31	63 867	38.20	62 933	35.29	35 571	22.67	98 504	29.39		
≥25 and <30	33 247	35.00	35 605	49.31	68 852	41.20	66 605	37.35	76 621	48.84	143 226	42.73		
≥30	18 009	18.96	15 122	20.94	33 131	19.82	46 288	25.96	42 810	27.29	89 098	26.58		
*Missing*	*228*	*0.24*	*184*	*0.25*	*412*	*0.25*	*1231*	*0.69*	*1464*	*0.93*	*2695*	*0.80*		
Smoking status, *n* %														
Never	58 250	61.38	38 345	53.11	96 595	57.77	103 772	58.19	73 108	46.60	176 880	52.77	*χ^2^* = 1583 *df* = 3 *P* <.001	*χ^2^* = 1491 *df* = 3 *P* <.001
Previous	30 716	32.33	27 592	38.22	58 308	34.87	54 713	30.68	60 003	38.25	114 716	34.22		
Current	5805	6.11	6078	8.42	11 883	7.11	18 556	10.41	22 523	14.36	41 079	12.25		
Prefer not to answer	182	0.19	156	0.22	338	0.20	880	0.49	839	0.53	1719	0.51		
*Missing*	*45*	*0.05*	*30*	*0.04*	*75*	*0.04*	*406*	*0.23*	*412*	*0.26*	*818*	*0.24*		
Participants, *n* %	94 998	56.82	72 201	43.18	167 199	100.00	178 327	53.20	156 885	46.80	335 212	100.00		
Alcohol frequency, *n* %														
Daily/almost daily	17 970	18.92	20 431	28.30	38 401	22.97	25 886	14.52	37 466	23.88	63 352	18.90	*χ^2^* = 3969 *df* = 6 *P* <.001	*χ^2^* = 2186 *df* = 6 *P* <.001
3-4 times/week	22 653	23.85	20 964	29.04	43 617	26.09	33 232	18.64	38 573	24.59	71 805	21.42		
1-2 times/week	24 472	25.76	17 588	24.36	42 060	25.16	45 692	25.62	41 519	26.46	87 211	26.02		
1-3 times/month	12 251	12.90	6136	8.50	18 387	11.00	23 243	13.03	14 210	9.06	37 453	11.17		
Special occasions only	11 470	12.07	3950	5.47	15 420	9.22	29 674	16.64	12 902	8.22	42 576	12.70		
Never	6105	6.43	3077	4.26	9182	5.49	19 938	11.18	11 507	7.33	31 445	9.38		
Prefer not to answer	32	0.03	24	0.03	56	0.03	254	0.14	293	0.19	547	0.16		
*Missing*	*45*	*0.05*	*31*	*0.04*	*76*	*0.05*	*408*	*0.23*	*415*	*0.26*	*823*	*0.25*		
Physical activity (IPAQ), *n* %														
Low	13 929	14.66	12 143	16.82	26 072	15.59	25 443	14.27	24 678	15.73	50 121	14.95	*χ^2^* = 137 *df* = 2 *P* <.001	*χ^2^* = 229 *df* = 2 *P* <.001
Moderate	34 732	36.56	25 916	35.89	60 648	36.27	55 774	31.28	47 577	30.33	103 351	30.83		
High	29 322	30.87	25 709	35.61	55 031	32.91	51 553	28.91	55 527	35.39	107 080	31.94		
*Missing*	*17 015*	*17.91*	*8433*	*11.68*	*25 448*	*15.22*	*45 557*	*25.55*	*29 103*	*18.55*	*74 660*	*22.27*		

A/AS Level, Advanced/Advanced Subsidiary Level; BMI, body mass index; CSE, Certificate of Secondary Education; HNC, Higher National Certificate; HND, Higher National Diploma; IPAQ, International Physical Activity Questionnaire; NVQ, National Vocational Qualification; O/GCSE, Ordinary/General Certificate of Education.

a Pretax household income; GBP, British pounds sterling.

### Health survey of England 2014

The HSE2014 contained 7871 adult responses (6904 [87.7%] with complete EQ-5D response data; frequencies of HSE2014 respondents by age, sex, and EQ-5D response completeness are shown in Table S3.

### EQ-5D descriptive system

#### UK Biobank EoPQ respondents

Descriptive dimension results are shown in Appendix S3 (Tables S4 and S5, and Figure S2).

Other than for *anxiety/depression*, the odds of reporting problems consistently increased with age, more so for males than for females. The maximal between-sex difference was in the *mobility* dimension at age >74 years (females OR = 2.35 [95% CI, 2.22-2.50]; males OR = 2.78 [95% CI, 2.57-2.99]). There were sex differences for the *self-care* and *pain/discomfort* domains for age group 55-64 years, with increased odds of reporting problems in these dimensions only evident for males. For the *anxiety/depression* dimension, compared with the reference group, the odds of reporting problems were lower for all age groups ≥55 years, with males also having consistently lower odds of reporting problems than females in each age group (Table S5).

#### Expected population norms

Descriptive dimensions results are shown in Appendix S3 (Tables S6-S8, and Figure S3).

For females, the odds of reporting problems increased significantly with age across all dimensions except for *anxiety/depression*. For males, the odds of reporting problems also increased consistently with age for *mobility* and *pain-discomfort*. For *self-care,* an increased odds of reporting problems was only evident in the oldest age group (>74 years, OR = 2.72 [95% CI, 1.67-4.43]) and a similar pattern was observed for *usual activities*, with increased odds only present for ages >65 years. For *anxiety/depression*, odds of reporting problems were lower than the reference for age group 65-74 years (OR = 0.66 [95% CI, 0.47-0.93]) with no other significant differences (Table S8).

#### Comparison of UK Biobank EoPQ respondents and expected population norms

**Figure 2:**
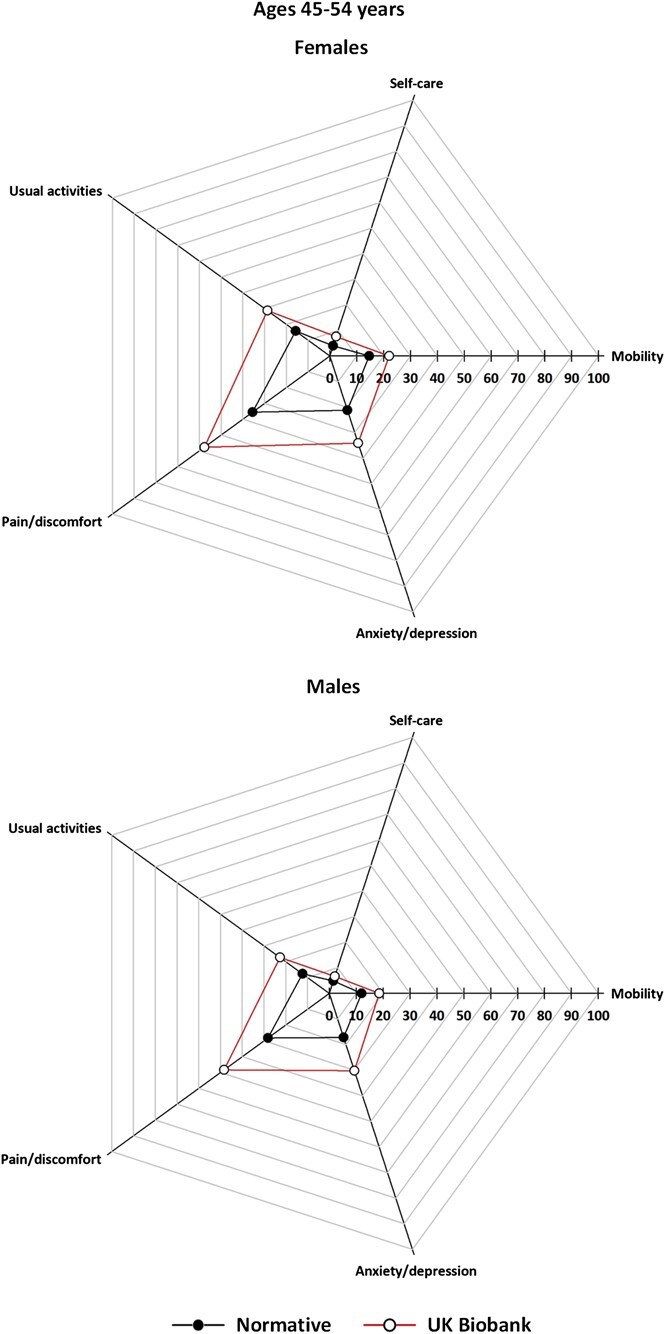
Prevalence of UK Biobank participants reporting problems of any severity, by EQ-5D descriptive dimension compared with population norms, age group 45-54 years, by sex; axis is the proportion of participants reporting a problem of any severity; that is, points move toward the edge as the proportion with problems increases.

**Figure 3:**
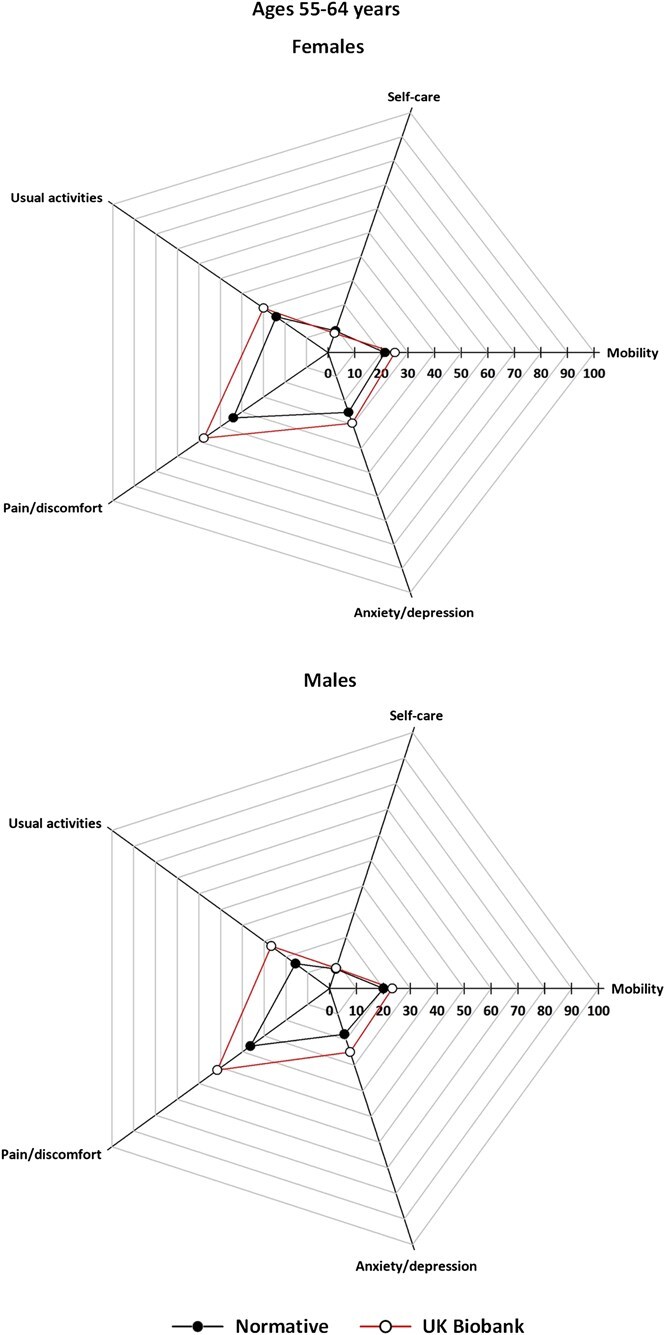
Prevalence of UK Biobank participants reporting problems of any severity, by EQ-5D descriptive dimension compared with population norms, age group 55-64 years, by sex; axis is the proportion of participants reporting a problem of any severity; that is, points move toward the edge as the proportion with problems increases.

**Figure 4:**
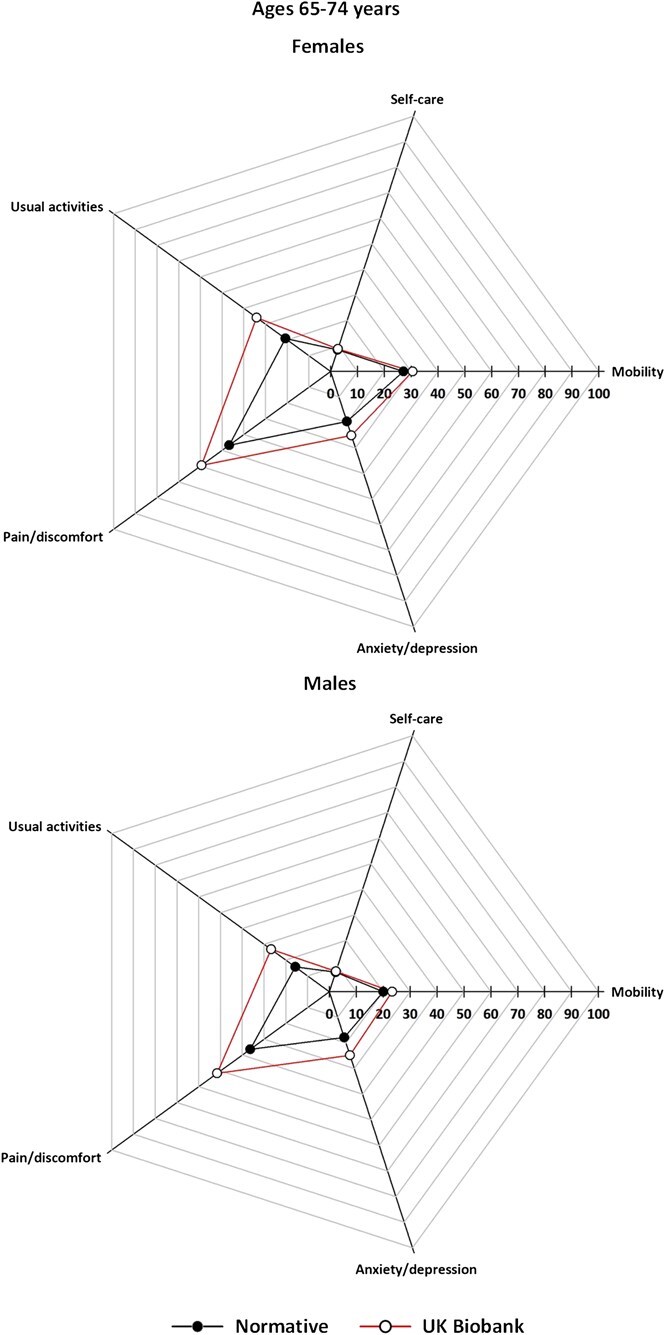
Prevalence of UK Biobank participants reporting problems of any severity, by EQ-5D descriptive dimension compared with population norms, age group 65-74 years, by sex; axis is the proportion of participants reporting a problem of any severity; that is, points move toward the edge as the proportion with problems increases.

**Figure 5:**
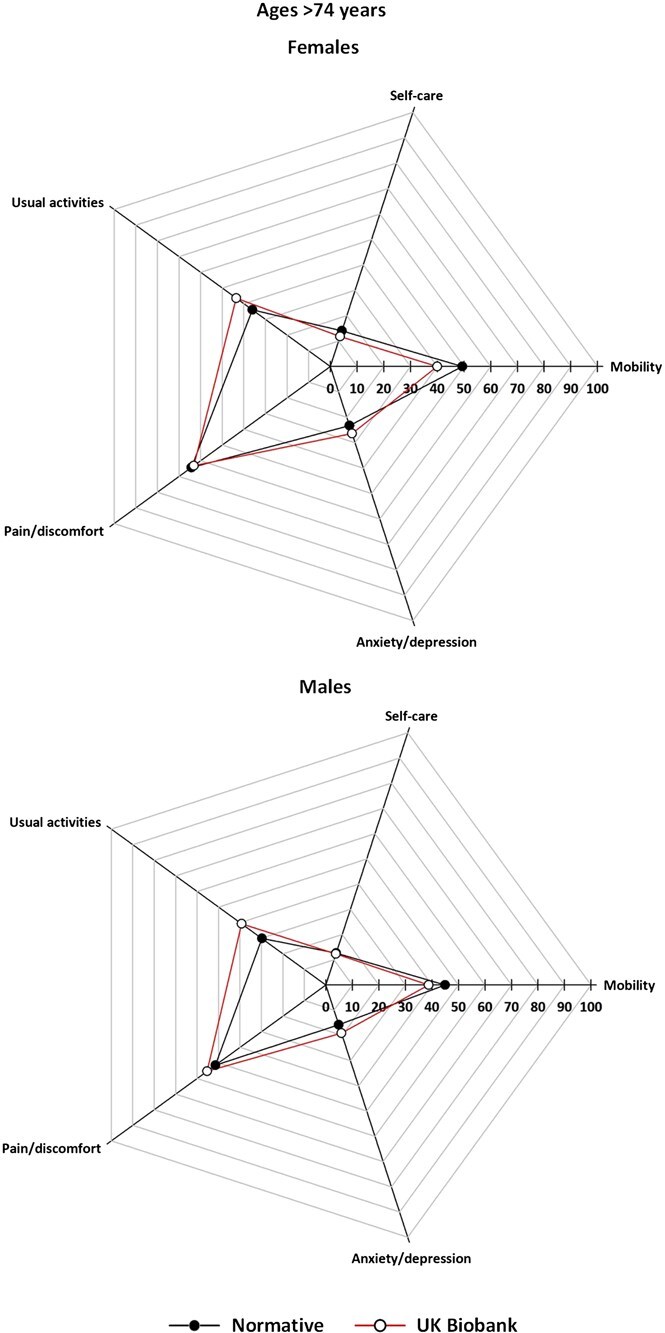
Prevalence of UK Biobank participants reporting problems of any severity, by EQ-5D descriptive dimension compared with population norms, age >74 years, by sex; axis is the proportion of participants reporting a problem of any severity; that is, points move toward the edge as the proportion with problems increases.

Figures 2-5 (data tabulated in Tables S5 and S8) show the proportion of EoPQ participants reporting problems compared to the expected population norms by age group and sex. The ORs/95% CIs are shown in Table 3; proportions are also presented in grouped bar charts to allow visualization of change within dimension by age (Figure S4).

**Table 3:** Counts, odds ratios (ORs)/95% CIs of reporting problems of any severity by EQ-5D dimension, UK Biobank participants, and expected population norm estimates by sex and age group.

	Females								Males							
Age	45-54		55-64		65-74		>74		45-54		55-64		65-74		>74	
	UK Biobank	Norm	UK Biobank	Norm	UK Biobank	Norm	UK Biobank	Norm	UK Biobank	Norm	UK Biobank	Norm	UK Biobank	Norm	UK Biobank	Norm
Mobility																
Some problems	1756	96	7666	114	12 893	122	5754	174	1029	77	4669	100	9690	106	5437	121
No problems	6188	558	22 823	419	29 298	327	8620	178	4505	564	15 325	398	22 969	302	8577	148
*Total*	*7944*	*654*	*30 489*	*533*	*42 191*	*449*	*14 374*	*352*	*5534*	*641*	*19 994*	*498*	*32 659*	*408*	*14 014*	*269*
OR (95% CI)	**1.65 (1.32-2.06)**		1.23 (1.00-1.52)		1.18 (0.96-1.45)		**0.68 (0.55-0.84)**		**1.67 (1.31-2.14)**		1.21 (0.97-1.51)		1.20 (0.96-1.50)		**0.78 (0.61-0.99)**	
Self-care																
Some problems	599	26	2439	48	3720	38	1693	49	366	31	1587	38	3177	31	1723	34
No problems	7345	627	28 050	484	38 471	411	12 681	300	5168	607	18 407	460	29 482	372	12 291	235
*Total*	*7944*	653	*30 489*	532	*42 191*	449	*14 374*	349	*5534*	638	*19 994*	498	*32 659*	403	*13 014*	269
OR (95% CI)	**1.97 (1.32-2.93)**		0.88 (0.65-1.18)		1.05 (0.75-1.46)		0.82 (0.60-1.11)		1.39 (0.95-2.02)		1.04 (0.75-1.46)		1.29 (0.90-1.86)		0.97 (0.68-1.39)	
Usual activities																
Some problems	2278	103	9138	129	14 409	94	6259	127	1255	79	5355	78	10 094	73	5503	79
No problems	5666	550	21 351	406	27 782	357	8115	225	4279	560	14 639	420	22 565	334	8511	186
*Total*	*7944*	653	*30 489*	535	*42 191*	451	14 374	352	*5534*	639	*19 994*	498	*32 659*	407	*14 014*	265
OR (95% CI)	**2.15 (1.73-2.66)**		**1.35 (1.10-1.64)**		**1.97 (1.57-2.47)**		**1.37 (1.10-1.70)**		**2.08 (1.63-2.65)**		**1.97 (1.54-2.51)**		**2.05 (1.59-2.64)**		**1.52 (1.17-1.98)**	
Pain/discomfort																
Some problems	4585	233	17 596	236	25 102	209	9078	227	2677	180	10 312	181	17 320	171	7757	138
No problems	3359	422	12 893	300	17 089	238	5296	126	2857	458	9682	317	15 339	237	6257	130
*Total*	*7944*	655	*30 489*	536	*42 191*	447	14 374	353	*5534*	638	*19 994*	498	*32 659*	408	*14 014*	268
OR (95% CI)	**2.47 (2.09-2.92)**		**1.73 (1.46-2.06)**		**1.67 (1.39-2.02)**		0.95 (0.77-1.19)		**2.38 (1.99-2.85)**		**1.87 (1.55-2.24)**		**1.56 (1.28-1.91)**		1.17 (0.92-1.49)	
Anxiety/depression																
Some problems	2708	138	8986	133	10 586	88	3793	81	1673	110	4969	89	6095	51	2677	42
No problems	5236	513	21 503	401	31 605	360	10 581	267	3861	528	15 025	409	26 564	358	11 337	224
*Total*	*7944*	651	*30 489*	534	*42 191*	448	14 374	348	*5534*	638	*19 994*	498	*32 659*	409	*14 014*	266
OR (95% CI)	**1.92 (1.58-2.33)**		**1.26 (1.03-1.53)**		**1.37 (1.08-1.73)**		1.18 (0.92-1.52)		**2.08 (1.68-2.57)**		**1.52 (1.21-1.91)**		**1.61 (1.20-2.16)**		1.26 (0.90-1.75)	

Statistically significant results shown in bold.

For all ages and both sexes, across the descriptive dimensions, we found consistently similar *patterns* in reported problems in the UKB and the normative data (Figures 2-5). However, there were notable differences in prevalence, with differences apparent in all age groups, but smallest for ages >74 years.

For ages 45-54 years there were clear UKB-norm differences in all dimensions for both sexes, except in the domain of *self-care*. Odds of reporting problems (reference, normative data) were similar for females and males and ranged from OR = 1.65 (95% 1.32-2.06) (females) and OR = 1.67 (95% CI, 1.31-2.14) (males) for *mobility* to OR = 2.47 (95% CI, 2.09-2.92) (females) and OR = 2.38 (95% CI, 1.99-2.85) (males) for *pain/discomfort*, this being the largest difference noted across all dimension comparisons (Figure 2, Table 3).

At ages 55-64 and 65-74 years there were UKB-norm differences for *usual activities*, *pain/discomfort,* and *anxiety/depression* (both sexes), but not in *mobility* or *self-care*. The ORs were consistently smaller for females than for males at ages 55-64 years. For females the largest OR was for *pain/discomfort* (OR = 1.73 [95% CI, 1.46-2.06]), whereas for males it was for *usual activities* (OR = 1.97 [95% CI, 1.54-2.51]) (Figure 4, Table 3). At ages 65-74 years the largest UKB-norm differences were for *usual activities* (females OR = 1.97 [95% CI, 1.57-2.47]; males OR = 2.05 [95% CI, 1.59-2.64]) (Figure 4, Table 3).

At age >74 years there were UKB-norm differences for both *mobility* and *usual activities* (both sexes); for *mobility* dimension, the UKB odds of reporting problems was lower than for the norms—the only such cases in all comparisons conducted (females OR = 0.68 [95% CI, 0.55-0.84], males OR = 0.78 [95% CI, 0.61-0.99]). For *usual activities*, for both sexes, the UKB odds of reporting problems were higher than for the norms (females OR = 1.37 [95% CI, 1.10-1.70], males OR = 1.52 [95% CI, 1.17-1.98]) (Figure 5, Table 3).

### EQ-5D health utilities

#### EoPQ respondents

Age-group and sex-specific utilities are presented in Appendix S4. EQ-5D-5L utilities (Devlin *et al*. (2018) method) are presented in Table S9 and Figure S5. These utilities are systematically higher than the utilities derived using the van Hout *et al*. (2012) (Table S10 and Figure S6) and Hernandez Alava *et al*. (2022) methods (Table S11 and Figure S7). Utilities derived using the latter 2 methods were very similar. For both sexes, utilities were similar across age groups, with a small decline seen for participants in the >74-year age group.

#### Expected population norms

##### Comparison of EoPQ respondents and expected population norms

**Figure 6:**
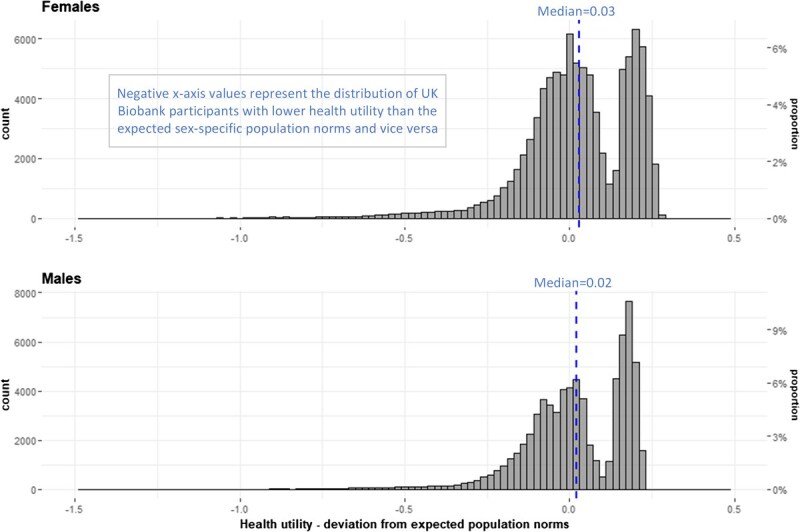
UK Biobank participants—deviation from expected EQ index population norms, counts, and proportions by sex. All age groups combined.

The distributions of deviations of UKB utilities from the expected population norm estimates were bimodal and deviations from zero were not statistically tested (Figure 6).

**Figure 7:**
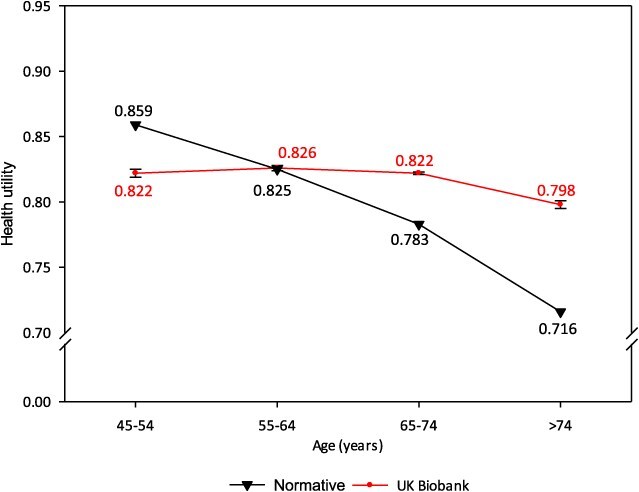
Mean health utility for females by age group, UK Biobank and expected population norms (van Hout 2012 crosswalk).

**Figure 8:**
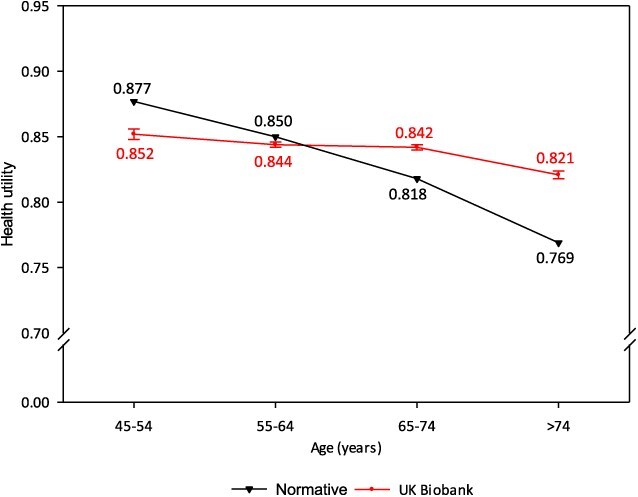
Mean health utility for males by age group, UK Biobank and expected population norms (van Hout 2012 crosswalk).

Comparisons of utilities, based on modeled age group midpoints, are shown in Figures 7 and 8.

At ages 45-54 years (both sexes), UKB participants had a modestly lower mean utility than the expected population norms (females –0.037; males –0.025). The UKB utilities then converged with the norms at age group 55-64 years, and subsequently diverged, with the normative utilities then showing a marked decline with increasing age (a reduction in mean utility of –0.143 (females) and –0.108 (males) comparing ages 45-54 and >74 years). In contrast, the decline in mean utility for UKB participants across age groups was very small (–0.024 females, –0.031 males). The largest departures from expected norms were in the >74-year age group (+0.082 females, +0.052 males).

While the absolute differences in UKB mean utilities and population norms were not large, the patterns of decline in utility with increasing age were markedly different, evident from the slopes in Figures 7 and 8.

## Discussion

For the first time, we examined the population-representativeness of a subset of the UKB using a multidimensional self-reported HRQoL lens. We build on previous studies that explored representativeness from the perspective of individual health determinants, such as socioeconomic, lifestyle, and environmental characteristics and risk factors.^5^^,^^8^ We found differences between the HRQoL of UKB participants and expected population norms and evidence suggestive of a healthy volunteer effect. We also identified that the EoPQ follow-up sample, from which the EQ-5D data were drawn, had more favorable baseline characteristics than other UKB participants.

### Representativeness of the EoPQ follow-up subsample from a health determinants perspective

Compared with the non-EoPQ subsample (*n* = 335 212), the EoPQ subsample (*n* = 167 199; ~51% response rate) was characterized by less ethnic diversity, with almost all participants identifying as White (a particular concern given the increasing ethnic diversity and ethnic inequalities among older adults in the United Kingdom),^39^^-^^41^ a higher level of education, higher income, more people in paid employment, less deprivation, less overweight or obesity, greater nonsmoking, more participation in moderate physical activity, and a higher proportion of people living without comorbidities. In contrast to these favorable characteristics, the EoPQ had a higher proportion of participants reporting regular alcohol consumption.

These subsample differences in health determinants are important and consistent with a prior study that found that UKB participants had sociodemographic characteristics and lifestyle risk factors that favored better health compared to the general UK population.^5^ A second study extended this approach and compared the characteristics of a subsample of participants who completed a UKB follow-up survey (mental health questionnaire, MHQ, 2016)^10^ with the UKB as a whole, and with HSE and census data. This study also found that the MHQ subsample was better educated, had higher socioeconomic status, and better health compared with the UKB as a whole, and the general UK population.^8^ Building on these studies, we have shown that the EoPQ follow-up subsample is also unrepresentative of the UKB sample as a whole, and hence even less representative of the wider population. These are important considerations for users of the EoPQ data.

### Health-related quality of life

For all HRQoL descriptive dimensions, all age groups, and for both sexes, the *patterns* of reported problems in the UKB closely followed those of expected population norms. That is, where a high or low proportion of UKB participants reported having problems for a given dimension, a high or low proportion was similarly evident in the normative data. However, while these patterns were similar, the prevalence of problems did vary; differences were most evident in the 45- to 54-year age group, with the largest differences being for the *pain/discomfort* dimension (females, ~60% UKB-reported problems vs <~40% in the norms; males, 50% UKB vs ~30% norms). Across all dimensions, for both sexes, the differences in prevalence narrowed with increasing age; for ages >74 years the proportions of UKB participants reporting problems were comparable with expected population norms. While a previous study found differences in the *self-care* dimension by ethnicity,^40^ this was not evident in our results, which is consistent with the UKB, and the EoPQ, being poorly representative of the ethnic diversity in the UK population.

UKB EoPQ utilities are different to the expected population norms. The norms showed a marked decrease in utilities with increasing age; this was expected and is consistent with patterns shown in published norms for other developed countries.^42^ However, EoPQ utilities did not exhibit this expected pattern of decline. For illustration, a graphical comparison of the UKB utilities with a range of published norms for developed countries norms is shown in Figure S9.

While differences in the patterns of decline were evident, absolute differences between UKB participant utilities and population norms were small; the largest differences being in the >74-year age group, with UKB participants having higher utilities than the norms (females, +0.082; males +0.052); differences of 8% and 5%, respectively. In all other age groups, the maximal deviations from expected norms were all <5% [females ages 65-74 years (+0.039); males ages 45-54 years (–0.025)].

The marked absence of decline in UKB utilities with increasing age appears consistent with healthy volunteer bias; this is not an unexpected finding, given the favorable baseline characteristics of EoPQ respondents.

### Strengths and limitations

We extended previous health determinant-focused studies by comparing the HRQoL, that is, participants’ self-reported health status, an output rather than a determinant, with population norms, and in doing so provide new information about representativeness that will be useful to users of the UKB EoPQ data generally, and the EQ-5D data specifically.

A strength of the work was our use of the EQ-5D multiattribute instrument for comparisons with expected population norms. This complements previous work that examined individual health determinants, by using a multidimensional measure of self-reported health status. We also reported health utilities derived using a set of commonly used valuation methods, increasing its usefulness to other researchers using the UKB EQ-5D data.

The work has some limitations: first, HRQoL data were not collected at the UKB baseline assessments and so it is not possible to draw any conclusions about the HRQoL of the UKB as a whole, only for the subset of participants who responded to the EoPQ. Second, we did not have access to HSE data for 2018 to allow the estimation of contemporaneous population norms for the UKB data. In the absence of an EQ-5D value set for England/United Kingdom, we also relied on instrument crosswalks and a 1997 EQ-5D-3L value set for England. Both of these data predate the EoPQ data by some years; these analyses could be revisited with new population norms and EQ-5D value sets as they become available.

## Conclusion

Extending previous knowledge about the representativeness of the UKB, we found that participants in the EoPQ follow-up survey had more favorable socioeconomic and health-related characteristics compared to both the UKB as a whole, and the wider UK population. However, when comparing HRQoL with expected population norms, we did not find large absolute differences and this should reassure users of the data; as per previous studies, we did find evidence suggestive of healthy volunteer bias.

### Ethics declaration

All participants provided written informed consent, and the UK Biobank has full ethical approval (NHS National Ethics Service; reference 11/NW/0382; 17/6/2011).

## Supplementary Material

Web_Material_kwaf113

## Data Availability

Terms of use of the Health Survey England, and the UK Biobank prevent us from sharing data, however both sources are available to approved bona fide researchers.

## References

[1] Allen NE, Lacey B, Lawlor DA, et al. Prospective study design and data analysis in UK Biobank. *Sci Transl Med*. 2024;16(729):eadf4428. 10.1126/scitranslmed.adf4428 38198570 PMC11127744

[2] Sudlow C, Gallacher J, Allen N, et al. UK biobank: an open access resource for identifying the causes of a wide range of complex diseases of middle and old age. *PLoS Med*. 2015;12(3):e1001779. 10.1371/journal.pmed.1001779 25826379 PMC4380465

[3] Brayne C, Moffitt TE. The limitations of large-scale volunteer databases to address inequalities and global challenges in health and aging. *Nat Aging*. 2022;2(9):775-783. 10.1038/s43587-022-00277-x 37118500 PMC10154032

[4] Catalogue of Bias Collaboration, Brassey J, Mahtani KR, et al. Volunteer bias. Catalogue Of Bias. 2017. Accessed August 1, 2024. http://www.catalogofbias.org/biases/volunteer-bias

[5] Fry A, Littlejohns TJ, Sudlow C, et al. Comparison of sociodemographic and health-related characteristics of UK Biobank participants with those of the general population. *Am J Epidemiol*. 2017;186(9):1026-1034. 10.1093/aje/kwx246 28641372 PMC5860371

[6] Keyes KM, Westreich D. UK Biobank, big data, and the consequences of non-representativeness. *Lancet*. 2019;393(10178):1297. 10.1016/S0140-6736(18)33067-8 PMC7825643 30938313

[7] Lindsted KD, Fraser GE, Steinkohl M, et al. Healthy volunteer effect in a cohort study: temporal resolution in the Adventist health study. *J Clin Epidemiol*. 1996;49(7):783-790. 10.1016/0895-4356(96)00009-1 8691229

[8] Davis KA, Coleman JR, Adams M, et al. Mental health in UK Biobank–development, implementation and results from an online questionnaire completed by 157 366 participants: a reanalysis. *BJPsych open*. 2020;6(2):e18. 10.1192/bjo.2019.100 32026800 PMC7176892

[9] NHS England . *Health Survey for England*. Accessed August 1, 2024. https://digital.nhs.uk/data-and-information/areas-of-interest/public-health/health-survey-for-england---health-social-care-and-lifestyles

[10] UK Biobank . *Thoughts and Feelings Questionnaire.* Accessed August 1, 2024. https://www.ukbiobank.ac.uk/explore-your-participation/contribute-further/thoughts-and-feelings-questionnaire

[11] Van Wilder L, Rammant E, Clays E, et al. A comprehensive catalogue of EQ-5D scores in chronic disease: results of a systematic review. *Qual Life Res*. 2019;28(12):3153-3161. 10.1007/s11136-019-02300-y 31531840

[12] Brooks R . EuroQol: the current state of play. *Health Policy*. 1996;37(1):53-72. 10.1016/0168-8510(96)00822-6 10158943

[13] Herdman M, Gudex C, Lloyd A, et al. Development and preliminary testing of the new five-level version of EQ-5D (EQ-5D-5L). *Qual Life Res*. 2011;20(10):1727-1736. 10.1007/s11136-011-9903-x 21479777 PMC3220807

[14] Janssen M, Pickard AS, Golicki D, et al. Measurement properties of the EQ-5D-5L compared to the EQ-5D-3L across eight patient groups: a multi-country study. *Qual Life Res*. 2013;22(7):1717-1727. 10.1007/s11136-012-0322-4 23184421 PMC3764313

[15] UK Biobank . *Pain Questionnaire*. 2022. Accessed August 1, 2024. https://biobank.ctsu.ox.ac.uk/crystal/ukb/docs/pain_questionnaire.pdf

[16] UK Biobank . About Us page. Accessed August 1, 2024, https://www.ukbiobank.ac.uk/learn-more-about-uk-biobank

[17] World Health Organisation . *A healthy lifestyle-WHO recommendations*. Accessed August 1, 2024, https://www.who.int/europe/news-room/fact-sheets/item/a-healthy-lifestyle---who-recommendations.

[18] Townsend P, Phillimore P, Beattie A. Health and deprivation: inequality and the North. London: Routledge; 2023.

[19] Craig CL, Marshall AL, Sjöström M, et al. International physical activity questionnaire: 12-country reliability and validity. *Med Sci Sports Exerc*. 2003;35(8):1381-1395. 10.1249/01.MSS.0000078924.61453.FB 12900694

[20] World Health Organization . International Statistical Classification of Diseases and Related Health Problems. 9th ed. 1978. Accessed August 1, 2024. World Health Organization (WHO). https://icd.who.int/

[21] World Health Organization . International Statistical Classification of Diseases and Related Health Problems. 10th ed. 1990. Accessed August 1, 2024. https://icd.who.int/

[22] Charlson ME, Pompei P, Ales KL, et al. A new method of classifying prognostic comorbidity in longitudinal studies: development and validation. *J Chronic Dis*. 1987;40(5):373-383. 10.1016/0021-9681(87)90171-8 3558716

[23] Bensken W. *Multimorbidity: Harmonizing Various Comorbidity, Multimorbidity, and Frailty Measures.* R package version 0.5.1. 2023. Accessed August 1, 2024. https://CRAN.R-project.org/package=multimorbidity

[24] EuroQoL Research Foundation . *EQ-5D User Guides*. Accessed August 1, 2024. https://euroqol.org/information-and-support/documentation/user-guides/

[25] National Institute for Health and Care Excellent (NICE) . *Glossary*. Accessed August 1, 2024. https://www.nice.org.uk/glossary

[26] Drummond MF, Sculpher MJ, Claxton K, et al. Methods for the Economic Evaluation of Health Care Programmes. Oxford University Press; 2015.

[27] McNamara S, Schneider PP, Love-Koh J, et al. Quality-adjusted life expectancy norms for the English population. *Value Health*. 2023;26(2):163-169. 10.1016/j.jval.2022.07.005 35965226

[28] Bridges S, Darton R, Evans-Lacko S, et al. Health Survey for England 2014 (HSE 2014), Volume 2: Methods and Documentation (Eds. Craig R, Fuller E, Mindell J). 2014. Accessed August 1, 2024. NHS England. https://digital.nhs.uk/data-and-information/publications/statistical/health-survey-for-england/health-survey-for-england-2014

[29] Devlin NJ, Shah KK, Feng Y, et al. Valuing health-related quality of life: an EQ-5 D-5 L value set for England. *Health Econ*. 2018;27(1):7-22. 10.1002/hec.3564 28833869 PMC6680214

[30] Alava MH, Pudney S, Wailoo A. The EQ-5D-5L value set for England: findings of a quality assurance program. *Value Health*. 2020;23(5):642-648. 10.1016/j.jval.2019.10.017 32389230

[31] van Hout B, Mulhern B, Feng Y, et al. The EQ-5D-5L value set for England: response to the “quality assurance”. *Value Health*. 2020;23(5):649-655. 10.1016/j.jval.2019.10.013 32389231

[32] van Hout B, Janssen M, Feng Y-S, et al. Interim scoring for the EQ-5D-5L: mapping the EQ-5D-5L to EQ-5D-3L value sets. *Value Health*. 2012;15(5):708-715. 10.1016/j.jval.2012.02.008 22867780

[33] Hernandez Alava M, Pudney S, Wailoo A. Estimating the relationship between EQ-5D-5L and EQ-5D-3L: results from a UK population study. *Pharmacoeconomics*. 2023;41(2):199-207. 10.1007/s40273-022-01218-7 36449173 PMC9883358

[34] Dolan P . Modeling valuations for EuroQol health states. *Med Care*. 1997;35(11):1095-1108. 10.1097/00005650-199711000-00002 9366889

[35] Lumley T. *Survey: analysis of complex survey samples*. R package version 4.0. 2023. Accessed August 1, 2024. https://CRAN.R-project.org/package=survey

[36] Morton F, Nijjar JS. *eq5d: Methods for Analysing 'EQ-5D' Data and Calculating 'EQ-5D' Index Scores*. R package version 0.12.0. 2022. Accessed August 1, 2024. https://CRAN.R-project.org/package=eq5d.

[37] Patil I . Visualizations with statistical details: The'ggstatsplot'approach. *J Open Source Softw*. 2021;6(61):3167. 10.21105/joss.03167

[38] R Core Team . R: A Language and Environment for Statistical Computing. R Foundation for Statistical Computing. 2022*.* https://www.R-project.org/

[39] Jivraj S, Simpson L. Ethnic Identity and Inequalities in Britain: The Dynamics of Diversity. Policy Press; 2015.

[40] Watkinson RE, Sutton M, Turner AJ. Ethnic inequalities in health-related quality of life among older adults in England: secondary analysis of a national cross-sectional survey. *Lancet Public Health*. 2021;6(3):e145-e154. 10.1016/S2468-2667(20)30287-5 33516278

[41] Bécares L, Jivraj S, Simpson L. Which Ethnic Groups have the Poorest Health? Ethnic Identity and Inequalities in Britain: The Dynamics of Diversity. Nine: which Ethnic Groups have the Poorest Health? Policy Press; 2015:123-140.

[42] Janssen MF, Szende A, Cabases J, et al. Population norms for the EQ-5D-3L: a cross-country analysis of population surveys for 20 countries. *Eur J Health Econ*. 2019;20(2):205-216. 10.1007/s10198-018-0955-5 29445941 PMC6438939

