# Supplementary material for: Health-related quality of life in the UK Biobank Experience of Pain follow-up study: a comparison with general population norms
Source: Am J Epidemiol. 2025 May 27;194(10):2954–67. doi: 10.1093/aje/kwaf113 (PMC12527277; doi:10.1093/aje/kwaf113)
Supplement: Web_Material_kwaf113 [file web_material_kwaf113.docx]

**Supplementary Material**

Health related quality of life in the UK Biobank *Experience of Pain* follow-up study: a comparison with general population norms

**Authors: Armfield NR, Farrell SF, Gabbe BJ, Elphinston RA, Kosgallana S, Connelly LB, Sterling M.**

**Table of Contents**

| **Appendix** | **Pages** |
| --- | --- |
| Appendix S1. Overview of EuroQoL EQ-5D | 3-8 |
| Appendix S2. Data Sources | 9-10 |
| Appendix S3. EQ-5D descriptive system figures and Tables | 11-21 |
| Appendix S4. EQ-5D Health utilities | 22-27 |

**List of Figures**

Figure S1. Example EQ-5D-3L descriptive dimension scoring and mapping the resulting health state to an EQ-5D health utility.

Figure S2. UK Biobank - proportions of participants reporting some problems vs. no problems by EQ-5D-5L descriptive dimension, sex, and age category (n=167,199).

Figure S3. HSE2014 adults ≥45y - EQ5D descriptive dimensions, weighted proportions, by age-group, sex and dichotomised level of problem

Figure S4. Proportions of participants reporting some problems and no problems, UK Biobank and expected population norm estimates, by EQ-5D descriptive dimension

Figure S5. UK Biobank - EQ-5D-5L health utility by age and sex – means and 95% confidence intervals (n=167,199)

Figure S6. UK Biobank - van Hout crosswalked EQ-5D-3L utilities by age and sex – means and 95% confidence intervals (n=167,199)

Figure S7. UK Biobank - DSU method mapped EQ-5D-3L utilities by age and sex - – means and 95% confidence intervals (n=167,199)

Figure S8. HSE2014 adults - EQ-5D-3L index modelled by age and sex using Dolan the 1997 value set for the United Kingdom

Figure S9. Population utilities from a multi-country analysis (Janssen 2019), with UK Biobank EoPQ utilities overlaid

**List of Tables**

Table S1. EuroQoL EQ-5D-3L and EQ-5D-5L responses for the mobility dimension

Table S2. Variable sources, UK Biobank and HSE 2014

Table S3. HSE2014 respondents by age-group, sex, and completeness of EQ-5D responses

Table S4. UK Biobank - frequencies and proportions by EQ-5D-5L descriptive dimension, level of problem, sex, and age category (n=167,199)

Table S5. UK Biobank - frequencies, proportions and odds of having problems of any severity by sex, and age group (n=167,199)

Table S6. HSE2014 Adult females – EQ-5D descriptive dimensions, raw counts, weighted proportions, by age group and dichotomised level of problem

Table S7. HSE2014 Adult males – EQ-5D descriptive dimensions, raw counts, weighted proportions, by age group and dichotomised level of problem

Table S8. HSE2014 adults ≥45y – EQ-5D descriptive dimensions, weighted counts, proportions and odds, by age-group, sex and dichotomised level of problem

Table S9. UK Biobank - EQ-5D-5L health utility (Devlin 2018 method) by age and sex (n=167,199)

Table S10. UK Biobank - van Hout Crosswalked EQ-5D-3L utilities by age and sex (n=167,199)

Table S11. UK Biobank - DSU method mapped EQ-5D-3L utilities by age and sex (n=167,199)

Table S12. HSE2014 - estimated expected EQ-5D-3L EQ Index values (age 18y-90y) age and sex, using Dolan 1997 value set for the United Kingdom

**Appendix S1. Overview of EuroQoL EQ-5D**

Here we provide an overview of the EuroQoL EQ-5D instruments used in this study, explain how participant responses are scored and summarised, and clarify our use of terminology relating to HRQoL in general, and the EQ-5D specifically.

**Glossary of terms**

Selected specific definitions of relevance to this study have been included below. The full table of standard EQ-5D terminology is available online at (<https://euroqol.org/information-and-support/documentation/terminology/>); see also Brooks R, Boye KS, Slaap B. J Patient Rep Outcomes. 2020 Jul 3;4(1):52.

**HRQoL** Health-related Quality of life is defined by the World Health Organization as “An individual’s perception of their position in life in the context of the culture and value systems in which they live and in relation to their goals, expectations, standards and concerns.” (<https://www.who.int/toolkits/whoqol>)

**EQ-5D** A standardised measure of health-related quality of life developed by the EuroQol Group to provide a simple, generic questionnaire for use in clinical and economic appraisal or population health status surveys.

**EQ-5D-3L** Refers to either the EQ-5D-3L descriptive system or the EQ-5D-3L questionnaire.

**EQ-5D-3L descriptive system** Descriptive system for health-related quality of life states in adults, consisting of five dimensions (*mobility*, *self-care*, *usual activities*, *pain/discomfort*, *anxiety/depression*), each of which has three severity levels that are described by statements appropriate to that dimension.

**EQ-5D-3L questionnaire** Standard layout for recording an adult person’s current self-reported health state. Consists of a standard format for respondents to record their health state according to the EQ-5D-3L descriptive system and the EQ VAS.

**EQ-5D-5L** Refers to either the EQ-5D-5L descriptive system or the EQ-5D-5L questionnaire.

**EQ-5D-5L descriptive system** Descriptive system for health-related quality of life states in adults, consisting of five dimensions (*mobility*, *self-care*, *usual activities*, *pain/discomfort*, *anxiety/depression*), each of which has three severity levels that are described by statements appropriate to that dimension.

**EQ-5D-5L questionnaire** Standard layout for recording an adult’s current self-reported health state. Consists of a standard format for respondents to record their health state according to the EQ-5D-5L descriptive system and the EQ VAS.

**EQ VAS** (*not used in this study*) A standard vertical 20 cm visual analogue scale, used in recording an individual’s rating of their overall current health-related quality of life. The scale ranges from 100 (‘the best imaginable health state’ or ‘the best health state you can imagine’) to 0 (‘the worst imaginable health state’ or ‘the worst health you can imagine’).

**EQ-5D profile, health state** A description of a health state defined by one of the EQ-5D descriptive systems. This may be summarised by a series of five sentences, one for each dimension and stating the level within that dimension; or a label consisting of five ordinal numbers, one for each dimension (by convention, in the order these appear in the questionnaire), defining the severity level, where 1 means no problems.

**EQ-5D value, index, score, utility, health utility** The value attached to an EQ-5D profile according to a set of weights that reflect, on average, people’s preferences about how good or bad the state is. Values are anchored at 1 (full health) and 0 (a state as bad as being dead) as required by their use in economic evaluation. Values less than 0 represent health states regarded as worse than a state that is as bad as being dead. An EQ-5D value is also sometimes known as an ‘index’, ‘score’ or ‘utility’.

**EQ-5D value set, tariff** A list of the value for every possible EQ-5D profile within a given descriptive system. For example, a value set for the EQ-5D-5L shows a value for each of the 3125 states that are described by it. These values are usually calculated using an algorithm that assigns a score to each level in each dimension, sometimes including adjustments for interactions between the dimensions. As value sets represent the average values of a sample of people, for example the general public of a particular country, it is important to state which value set is being used. Value sets are also sometimes referred to as ‘tariffs’.

*Background*

The EQ-5D is a family of instruments developed by EuroQol (<https://euroqol.org/>). The instruments (simple, self-report questionnaires) ask respondents to rate their ‘health today’ and are used extensively in health research, clinically, in health-related economic decision making, and to assess population health.

EQ-5D instruments comprise two components: the *descriptive system* and the *EQ VAS* (the EQ VAS was not used in these analyses and is not described further).

In this study, we used data collected using the EQ-5D-3L and (the more recent) EQ-5D-5L instruments. EQ-5D-3L was used in the Health Survey for England (i.e. used by us to estimate expected population norms), and EQ-5D-5L was used by the UQ Biobank in the experience of pain questionnaire (EoPQ). The EQ-5D-3L and EQ-5D-5L are generic, that is they are not specific to a specific condition, disease or population group.

*The EQ-5D Descriptive system*

Within the descriptive system, EQ-5D instruments assess health across five dimensions (hence ‘EQ-5D’); these dimensions being *mobility*, *self-care*, *usual activities*, *pain/discomfort* and *anxiety/depression*. For each dimension, the respondent is asked to report the severity of any problems they may be experiencing.

Both the EQ-5D-3L and EQ-5D-5L ask questions about the same five dimensions of health described above. The instruments differ only in the levels of problem severity response options available. Specifically, the EQ-5D-3L allows the respondent to describe their problems according to three levels of severity (hence ‘3L’), while EQ-5D-5L provides for five levels of problem severity.

For example, in the *mobility* dimension, the available responses for each instrument are shown below in Table S1.

**Table S1. EuroQoL EQ-5D-3L and EQ-5D-5L responses for the mobility dimension (S1-1)**

| **Severity** | **EQ-5D-3L** | **Score** | **EQ-5D-5L** | **Score** |
| --- | --- | --- | --- | --- |
| *Lowest* | I have no problems in walking about | 1 | I have no problems in walking about | 1 |
|  | I have some problems in walking about | 2 | I have slight problems in walking about | 2 |
|  | I am confined to bed | 3 | I have moderate problems in walking about | 3 |
|  | N/A | N/A | I have severe problems in walking about | 4 |
| *Highest* | N/A | N/A | I am unable to walk  about | 5 |

*Procedures for scoring and summarisation of EQ-5D responses*

In this study, we followed the standard procedures for scoring and summarising responses as described in the EuroQoL EQ-5D user guides (<https://euroqol.org/information-and-support/documentation/user-guides>). For each dimension, responses are scored on a three-point scale (1, lowest severity to 3, highest severity) for EQ-5D-3L, and a five-point scale (1 to 5) for EQ-5D-5L (Table S1).

While there are other ways to generate summary scores, the standard EQ-5D approach to summarise a respondent’s, ‘health today’ is to catenate their response scores across all five dimensions. This summary value is variously described as a *health state*, *health profile*, or *health index* in the literature. In this paper we use *health state* to describe this summary value.

Figure S1 shows example scoring and derivation of the health state ‘21221’ for a hypothetical EQ-5D-3L respondent with *some* mobility problems (score 2), *no self-care problems* (1), some problems with *usual activities* (2) and *pain/discomfort* (2), and no problems with *anxiety/depression* (1)

EQ-5D-3L has 243 (3^5^) possible unique health states (‘11111’, *no problems* in any dimension to ‘33333’, *unable/extreme* in all dimensions); EQ-5D-5L has 3,125 (5^5^) possible states (‘11111’ to ‘55555’).

*From health states to health utilities*

Health states provide a standard way to summarise an individual’s health; i.e., individuals in different countries with the same EQ-5D responses would have identical summary health states. However, the *value or preference* that an individual or society gives to being in a particular health state varies by country. Using the previous example from above and Figure S1, on average people in the UK may value being in health state ‘21221’ quite differently to people in say Japan, or the USA, or India.

Country specific quantitative values of health states are represented using *health utilities*.^a^

Health utilities (also referred to as *EQ-5D value*)^b^ are represented by a decimal number; whereby a utility value of ‘1’ represents full health, ‘0’ is as bad as being dead, and values <0 represent a state worse than being dead.

Utilities are computed using weights from population-specific *value sets* (also referred to in the literature as *tariffs)*. The example shown in Figure S1 shows the translation of the ‘21221’ EQ-5D-3L *health state* to a *health utility* of 0.691 using to a published value set for the UK.

--

^a^National Institute for Health and Care Excellent (NICE). *Glossary*. Retrieved 1 Aug 2024, from <https://www.nice.org.uk/glossary>.

bWhile the economics literature uses the term ‘utility’ to describe health-related preferences which incorporate uncertainty (see Drummond, M. F., Sculpher, M. J., Claxton, K., Stoddart, G. L., & Torrance, G. W. (2015). Methods for the economic evaluation of health care programmes: Oxford university press), the general literature uses *health state value* and *health utility* interchangeably; here we have chosen to use *health utility* to be consistent with the usage within the EQ-5D literature which we cite.

**Figure S1. Example EQ-5D-3L descriptive dimension scoring and mapping the resulting health state to an EQ-5D health utility**
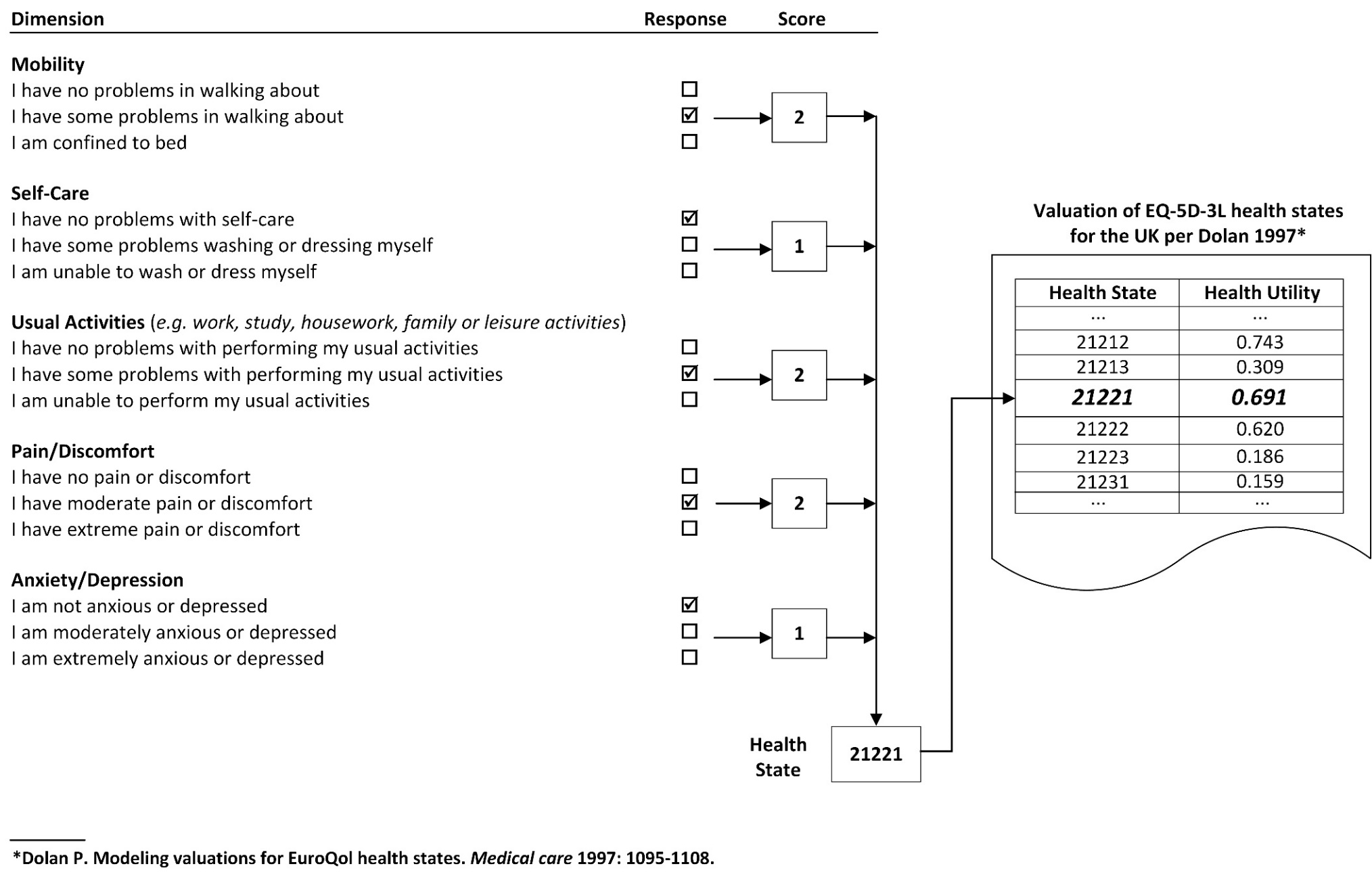


**Appendix S2. Data Sources**

**Table S2. Variable sources, UK Biobank and HSE 2014**

| **Data Item** | **UK Biobank**  **Data-field** | **HSE 2014**  **variable*** |
| --- | --- | --- |
| **Participant characteristics** |  |  |
| Age at recruitment <https://biobank.ndph.ox.ac.uk/showcase/field.cgi?id=21022> | 20122 | - |
| Sex  <https://biobank.ndph.ox.ac.uk/showcase/field.cgi?id=31> | 31 | - |
| Ethnic background <https://biobank.ndph.ox.ac.uk/showcase/field.cgi?id=21000> | 21000 | - |
| Highest level of education <https://biobank.ndph.ox.ac.uk/showcase/field.cgi?id=6138> | 6138 | - |
| Current employment status  <https://biobank.ndph.ox.ac.uk/showcase/field.cgi?id=6142> | 6142 |  |
| Pre-tax household income  <https://biobank.ndph.ox.ac.uk/showcase/field.cgi?id=738> | 738 |  |
| Townsend Deprivation Index (TDI) <https://biobank.ndph.ox.ac.uk/showcase/field.cgi?id=22189> | 22189 | - |
| Body Mass Index (BMI) <https://biobank.ndph.ox.ac.uk/showcase/field.cgi?id=21001> | 21001 | - |
| Smoking status <https://biobank.ndph.ox.ac.uk/showcase/field.cgi?id=20116> | 20116 | - |
| Alcohol frequency <https://biobank.ndph.ox.ac.uk/showcase/field.cgi?id=1558> | 1558 | - |

| Table S2 (contd). |  |  |
| --- | --- | --- |
| **Participant characteristics (contd.)** |  |  |
| Physical activity <https://biobank.ndph.ox.ac.uk/showcase/field.cgi?id=22032> | 22032 | - |
| **Linked hospital inpatient ICD-9 and ICD-10 data**  ICD-9  *Diagnosis codes*  <https://biobank.ndph.ox.ac.uk/showcase/field.cgi?id=41270>  *Dates of corresponding first inpatient diagnosis*  <https://biobank.ndph.ox.ac.uk/showcase/field.cgi?id=41280>  ICD-10  *Diagnosis codes*  <https://biobank.ndph.ox.ac.uk/showcase/field.cgi?id=41271>  *Dates of corresponding first inpatient diagnosis*  <https://biobank.ndph.ox.ac.uk/showcase/field.cgi?id=41281> | 41270  41280  41271  41281 | - |
| **EuroQoL EQ-5D item** |  |  |
| Descriptive system dimensions |  |  |
| *Mobility problems today*  <https://biobank.ndph.ox.ac.uk/showcase/field.cgi?id=120098> | 120098 | MOBILITY |
| *Self-care problems today*  <https://biobank.ndph.ox.ac.uk/showcase/field.cgi?id=120099> | 120099 | SELFCARE |
| *Problems doing usual activities*  <https://biobank.ndph.ox.ac.uk/showcase/field.cgi?id=120100> | 120100 | USUALACT |
| *Pain/discomfort today*  <https://biobank.ndph.ox.ac.uk/showcase/field.cgi?id=120101> | 120101 | PAIN |
| *Anxiety/depression today*  <https://biobank.ndph.ox.ac.uk/showcase/field.cgi?id=120102> | 120102 | ANXIETY |
| **Individual level survey weights** | - | WT_INT |

*** UK Data Archive Study Number 7919 - Health Survey for England 2014. List of Variables Version 1. [https://doc.ukdataservice.ac.uk/doc/7919/mrdoc/pdf/7919_hse2014_dataset_documents.pdf. 2014](https://doc.ukdataservice.ac.uk/doc/7919/mrdoc/pdf/7919_hse2014_dataset_documents.pdf.%202014)

**Table S3. HSE2014 respondents by age-group, sex, and completeness of EQ-5D responses**

| **Age Group (y)** | **<18** | **18-24** | **25-34** | **35-44** | **45-54** | **55-64** | **65-74** | **>=75** | *Total* | *Adults* |
| --- | --- | --- | --- | --- | --- | --- | --- | --- | --- | --- |
| **Total respondents** | | | | | | | | | | |
| Females | 1,071 | 310 | 672 | 804 | 816 | 642 | 640 | 501 | *5,456* | *4,385* |
| Males | 1,138 | 264 | 456 | 606 | 665 | 567 | 550 | 378 | *4,624* | *3,486* |
| *Total* | *2,209* | *574* | *1,128* | *1,410* | *1,481* | *1,209* | *1,190* | *879* | *10,080* | *7,871* |
|  | | | | | | | | | | |
| **Adults with complete EQ-5D responses*** | | | | | | | | | *Total* | *%* |
| Females |  | 270 | 606 | 724 | 733 | 584 | 578 | 385 | *3,880* | *88.5* |
| Males |  | 230 | 385 | 527 | 596 | 491 | 486 | 309 | *3,024* | *86.7* |
| *Total* |  | *500* | *991* | *1,251* | *1,329* | *1,075* | *1,064* | *694* | *6,904* | *87.7* |

**complete adult responses used for modelling of health utilities;* Bridges S, Darton R, Evans-Lacko S, et al. Health survey for England 2014 (HSE 2014), Volume 2: Methods and documentation (Eds. Craig R, Fuller E, Mindell J). *London: Joint Health Service Unit, NatCen Social Research Department of Epidemiology and Public Health, UCL*. 2014.

**Appendix S3. EQ-5D descriptive system figures and Tables**

**Table S4. UK Biobank - frequencies and proportions by EQ-5D-5L descriptive dimension, level of problem, sex, and age category (n=167,199)**

|  | **Females**  **(n=94,998)** | | | | | | | | **Males**  **(n=72,201)** | | | | | | | |
| --- | --- | --- | --- | --- | --- | --- | --- | --- | --- | --- | --- | --- | --- | --- | --- | --- |
| **Age** | 45-54 | | 55-64 | | 65-74 | | >74 | | 45-54 | | 55-64 | | 65-74 | | >74 | |
|  | *n=7,944* | | *n=30,489* | | *n=42,191* | | *n=14,374* | | *n=5,534* | | *n=19,994* | | *n=32,659* | | *n=14,014* | |
| **Mobility** | *n* | *%* | *n* | *%* | *n* | *%* | *n* | *%* | *n* | *%* | *n* | *%* | *n* | *%* | *n* | *%* |
| *None* | 6,188 | 77.90 | 22,823 | 74.86 | 29,298 | 69.44 | 8,620 | 59.97 | 4,505 | 81.41 | 15,325 | 76.65 | 22,969 | 70.33 | 8,577 | 61.20 |
| *Slight* | 1,203 | 15.14 | 5,192 | 17.03 | 8,228 | 19.50 | 3,273 | 22.77 | 767 | 13.86 | 3,309 | 16.55 | 6,409 | 19.62 | 3,259 | 23.26 |
| *Moderate* | 420 | 5.29 | 1,867 | 6.12 | 3,576 | 8.48 | 1,880 | 13.08 | 190 | 3.43 | 950 | 4.75 | 2,482 | 7.60 | 1,617 | 11.54 |
| *Severe* | 124 | 1.56 | 561 | 1.84 | 1,028 | 2.44 | 573 | 3.99 | 62 | 1.12 | 368 | 1.84 | 756 | 2.31 | 539 | 3.85 |
| *Unable* | 9 | 0.11 | 46 | 0.15 | 61 | 0.14 | 28 | 0.19 | 10 | 0.18 | 42 | 0.21 | 43 | 0.13 | 22 | 0.16 |
| Number/% reporting problems | 1,756 | 22.10 | 7,666 | 25.14 | 12,893 | 30.56 | 5,754 | 40.03 | 1,029 | 18.59 | 4,669 | 23.35 | 9,690 | 29.67 | 5,437 | 38.80 |
| **Self-care** |  |  |  |  |  |  |  |  |  |  |  |  |  |  |  |  |
| *None* | 7,345 | 92.46 | 28,050 | 92.00 | 38,471 | 91.18 | 12,681 | 88.22 | 5,168 | 93.33 | 18,407 | 92.06 | 29,482 | 90.27 | 12,291 | 87.71 |
| *Slight* | 423 | 5.32 | 1,739 | 5.70 | 2,736 | 6.48 | 1,270 | 8.84 | 265 | 4.79 | 1,151 | 5.76 | 2,465 | 7.55 | 1,324 | 9.45 |
| *Moderate* | 140 | 1.76 | 543 | 1.78 | 796 | 1.89 | 364 | 2.53 | 82 | 1.48 | 340 | 1.70 | 590 | 1.81 | 326 | 2.33 |
| *Severe* | 32 | 0.40 | 132 | 0.43 | 155 | 0.37 | 45 | 0.31 | 14 | 0.25 | 81 | 0.41 | 104 | 0.32 | 60 | 0.43 |
| *Unable* | 4 | 0.05 | 25 | 0.08 | 33 | 0.08 | 14 | 0.10 | 5 | 0.09 | 15 | 0.08 | 18 | 0.06 | 13 | 0.09 |
| Number/% reporting problems | 599 | 7.54 | 2,439 | 8.00 | 3,720 | 8.82 | 1,693 | 11.78 | 366 | 6.61 | 1,587 | 7.94 | 3,177 | 9.73 | 1,723 | 12.29 |
| **Usual activities** |  |  |  |  |  |  |  |  |  |  |  |  |  |  |  |  |
| *None* | 5,666 | 71.32 | 21,351 | 70.03 | 27,782 | 65.85 | 8,115 | 56.46 | 4,279 | 77.32 | 14,639 | 73.32 | 22,565 | 69.09 | 8,511 | 60.73 |
| *Slight* | 1,598 | 20.12 | 6,477 | 21.23 | 10,103 | 23.95 | 4,126 | 28.70 | 921 | 16.64 | 3,855 | 19.28 | 7,304 | 22.36 | 3,828 | 27.32 |
| *Moderate* | 493 | 6.21 | 2,023 | 6.64 | 3,414 | 8.09 | 1,724 | 11.99 | 249 | 4.50 | 1,120 | 5.60 | 2,218 | 6.79 | 1,333 | 9.51 |
| *Severe* | 157 | 1.98 | 522 | 1.71 | 745 | 1.77 | 356 | 2.48 | 69 | 1.25 | 309 | 1.55 | 487 | 1.49 | 271 | 1.93 |
| *Unable* | 30 | 0.38 | 116 | 0.38 | 147 | 0.35 | 53 | 0.37 | 16 | 0.29 | 71 | 0.36 | 85 | 0.26 | 71 | 0.51 |
| Number/% reporting problems | 2,278 | 28.68 | 9,138 | 29.97 | 14,409 | 34.15 | 6,259 | 43.54 | 1,255 | 22.68 | 5,355 | 26.78 | 10,094 | 30.91 | 5,503 | 39.27 |
| **Pain/Discomfort** |  |  |  |  |  |  |  |  |  |  |  |  |  |  |  |  |
| *None* | 3,359 | 42.28 | 12,893 | 42.29 | 17,089 | 40.50 | 5,296 | 36.84 | 2,857 | 51.63 | 9,682 | 48.42 | 15,339 | 46.97 | 6,257 | 44.65 |
| *Slight* | 3,254 | 40.96 | 12,685 | 41.61 | 17,957 | 42.56 | 6,071 | 42.24 | 2,122 | 38.34 | 7,927 | 39.65 | 13,330 | 40.82 | 5,702 | 40.69 |
| *Moderate* | 1,092 | 13.75 | 4,090 | 13.41 | 6,119 | 14.50 | 2,579 | 17.94 | 470 | 8.49 | 1,974 | 9.87 | 3,423 | 10.48 | 1,801 | 12.85 |
| *Severe* | 198 | 2.49 | 732 | 2.40 | 949 | 2.25 | 399 | 2.78 | 70 | 1.26 | 364 | 1.82 | 524 | 1.60 | 233 | 1.66 |
| *Extreme* | 41 | 0.52 | 89 | 0.29 | 77 | 0.18 | 29 | 0.20 | 15 | 0.27 | 47 | 0.24 | 43 | 0.13 | 21 | 0.15 |
| Number/% reporting problems | 4,585 | 57.72 | 17,596 | 57.71 | 25,102 | 59.50 | 9,078 | 63.16 | 2,677 | 48.37 | 10,312 | 51.58 | 17,320 | 53.03 | 7,757 | 55.35 |
| **Anxiety/Depression** |  |  |  |  |  |  |  |  |  |  |  |  |  |  |  |  |
| *None* | 5,236 | 65.91 | 21,503 | 70.53 | 31,605 | 74.91 | 10,581 | 73.61 | 3,861 | 69.77 | 15,025 | 75.15 | 26,564 | 81.34 | 11,337 | 80.90 |
| *Slight* | 1,997 | 25.14 | 6,823 | 22.38 | 8,433 | 19.99 | 3,059 | 21.28 | 1,224 | 22.12 | 3,745 | 18.73 | 4,880 | 14.94 | 2,202 | 15.71 |
| *Moderate* | 574 | 7.23 | 1,756 | 5.76 | 1,863 | 4.42 | 661 | 4.60 | 361 | 6.52 | 990 | 4.95 | 1,050 | 3.22 | 423 | 3.02 |
| *Severe* | 94 | 1.18 | 290 | 0.95 | 229 | 0.54 | 59 | 0.41 | 68 | 1.23 | 183 | 0.92 | 137 | 0.42 | 47 | 0.34 |
| *Extreme* | 43 | 0.54 | 117 | 0.38 | 61 | 0.14 | 14 | 0.10 | 20 | 0.36 | 51 | 0.26 | 28 | 0.09 | 5 | 0.04 |
| Number/% reporting problems | 2,708 | 34.09 | 8,986 | 29.47 | 10,586 | 25.09 | 3,793 | 26.39 | 1,673 | 30.23 | 4,969 | 24.85 | 6,095 | 18.66 | 2,677 | 19.10 |

**Table S5. UK Biobank - frequencies, proportions and odds of having problems of any severity by sex, and age group (n=167,199)**

|  | **Females**  **(n=94,998)** | | | | | | | | **Males**  **(n=72,201)** | | | | | | | |
| --- | --- | --- | --- | --- | --- | --- | --- | --- | --- | --- | --- | --- | --- | --- | --- | --- |
| **Age** | 45-54 | | 55-64 | | 65-74 | | >74 | | 45-54 | | 55-64 | | 65-74 | | >74 | |
|  | *n=7,944* | | *n=30,489* | | *n=42,191* | | *n=14,374* | | *n=5,534* | | *n=19,994* | | *n=32,659* | | *n=14,014* | |
| **Mobility** | *n* | *%* | *n* | *%* | *n* | *%* | *n* | *%* | *n* | *%* | *n* | *%* | *n* | *%* | *n* | *%* |
| No problems | 6,188 | 77.90 | 22,823 | 74.86 | 29,298 | 69.44 | 8,620 | 59.97 | 4,505 | 81.41 | 15,325 | 76.65 | 22,969 | 70.33 | 8,577 | 61.20 |
| Some problems | 1,756 | 22.10 | 7,666 | 25.14 | 12,893 | 30.56 | 5,754 | 40.03 | 1,029 | 18.59 | 4,669 | 23.35 | 9,690 | 29.67 | 5,437 | 38.80 |
| OR (95% CI) | *reference* | | **1.18 (1.12 - 1.26)** | | **1.55 (1.46 – 1.64)** | | **2.35 (2.21 – 2.50)** | | *reference* | | **1.33 (1.24 – 1.44)** | | **1.85 (1.72 – 1.98)** | | **2.78 (2.57 – 2.99)** | |
| **Self-care** |  |  |  |  |  |  |  |  |  |  |  |  |  |  |  |  |
| No problems | 7,345 | 92.46 | 28,050 | 92.00 | 38,471 | 91.18 | 12,681 | 88.22 | 5,168 | 93.33 | 18,407 | 92.06 | 29,482 | 90.27 | 12,291 | 87.71 |
| Some problems | 599 | 7.54 | 2,439 | 8.00 | 3,720 | 8.82 | 1,693 | 11.78 | 366 | 6.61 | 1,587 | 7.94 | 3,177 | 9.73 | 1,723 | 12.29 |
| OR (95% CI) | *reference* | | 1.07 (0.97 – 1.17) | | **1.19 (1.08 – 1.30)** | | **1.64 (1.48 – 1.80)** | | *reference* | | **1.22 (1.08 – 1.37)** | | **1.52 (1.36 – 1.70)** | | **1.98 (1.76 – 2.23)** | |
| **Usual activities** |  |  |  |  |  |  |  |  |  |  |  |  |  |  |  |  |
| No problems | 5,666 | 71.32 | 21,351 | 70.03 | 27,782 | 65.85 | 8,115 | 56.46 | 4,279 | 77.32 | 14,639 | 73.32 | 22,565 | 69.09 | 8,511 | 60.73 |
| Some problems | 2,279 | 28.69 | 9,138 | 29.97 | 14,409 | 34.15 | 6,259 | 43.54 | 1,255 | 22.68 | 5,355 | 26.78 | 10,094 | 30.91 | 5,503 | 39.27 |
| OR (95% CI) | *reference* | | **1.06 (1.01 – 1.12)** | | **1.29 (1.22 – 1.36)** | | **1.92 (1.81 – 2.03)** | | *reference* | | **1.25 (1.16 – 1.34)** | | **1.53 (1.43 – 1.63)** | | **2.20 (2.05 – 2.37)** | |
| **Pain/Discomfort** |  |  |  |  |  |  |  |  |  |  |  |  |  |  |  |  |
| No problems | 3,359 | 42.28 | 12,893 | 42.29 | 17,089 | 40.50 | 5,296 | 36.84 | 2,857 | 51.63 | 9,682 | 48.42 | 15,339 | 46.97 | 6,257 | 44.65 |
| Some problems | 4,585 | 57.72 | 17,596 | 57.71 | 25,102 | 59.50 | 9,078 | 63.16 | 2,677 | 48.37 | 10,312 | 51.58 | 17,320 | 53.03 | 7,757 | 55.35 |
| OR (95% CI) | *reference* | | 1.0 (0.95 – 1.05) | | **1.08 (1.03 – 1.13)** | | **1.26 (1.19 – 1.33)** | | *reference* | | **1.14 (1.07 – 1.21)** | | **1.21 (1.14 – 1.28)** | | **1.32 (1.24 – 1.41)** | |
| **Anxiety/Depression** |  |  |  |  |  |  |  |  |  |  |  |  |  |  |  |  |
| No problems | 5,236 | 65.91 | 21,503 | 70.53 | 31,605 | 74.91 | 10,581 | 73.61 | 3,861 | 69.77 | 15,025 | 75.15 | 26,564 | 81.34 | 11,337 | 80.90 |
| Some problems | 2,708 | 34.09 | 8,986 | 29.47 | 10,586 | 25.09 | 3,793 | 26.39 | 1,673 | 30.23 | 4,969 | 24.85 | 6,095 | 18.66 | 2,677 | 19.10 |
| OR (95% CI) | *reference* | | **0.81 (0.77 – 0.85)** | | **0.65 (0.62 – 0.68)** | | **0.69 (0.65 – 0.74)** | | *reference* | | **0.76 (0.71 – 0.82)** | | **0.53 (0.50 – 0.56)** | | **0.54 (0.51 – 0.59)** | |

**Figure S2. UK Biobank - proportions of participants reporting some problems vs. no problems by EQ-5D-5L descriptive dimension, sex, and age category (n=167,199)**





|  |
| --- |
| 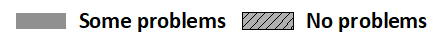 |

**Figure S2 (contd). UK Biobank - proportions of participants reporting some problems vs. no problems by EQ-5D-5L descriptive dimension, sex, and age category (n=167,199)**


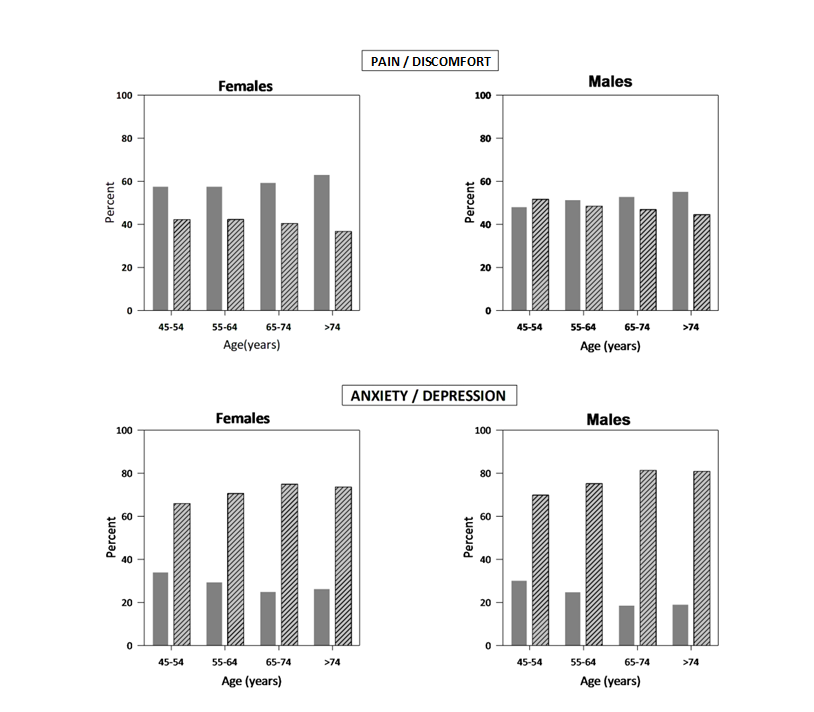


|  |  |
| --- | --- |
| 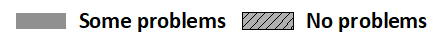 | |

**Table S6. HSE2014 Adult females – EQ-5D descriptive dimensions, raw counts, weighted proportions, by age group and dichotomised level of problem**

|  |  | **Age Group** | | | | | | | | | | | | | |
| --- | --- | --- | --- | --- | --- | --- | --- | --- | --- | --- | --- | --- | --- | --- | --- |
| **Total** |  | **18-24y** | | **25-34y** | | **35-44y** | | **45-54y** | | **55-64y** | | **65-74y** | | **>74y** | |
|  |  | *n* | *%* | *n* | *%* | *n* | *%* | *n* | *%* | *n* | *%* | *n* | *%* | *n* | *%* |
| Mobility | No problems | 261 | 95.61 | 560 | 92.98 | 659 | 89.53 | 636 | 85.32 | 463 | 78.61 | 427 | 72.83 | 202 | 50.57 |
|  | Some Problems | 13 | 4.39 | 45 | 6.86 | 74 | 10.31 | 104 | 14.53 | 126 | 21.39 | 161 | 27.17 | 203 | 49.43 |
|  | Confined to bed | 0 | 0 | 1 | 0.16 | 1 | 0.16 | 1 | 0.15 | 0 | 0 | 0 | 0 | 1 | 0.0 |
|  | *Total* | *274* | *100* | *606* | *100* | *734* | *100* | *741* | *100* | *589* | *100* | *588* | *100* | *406* | *100* |
|  | (Missing) | 36 |  | 66 |  | 70 |  | 75 |  | 53 |  | 52 |  | 95 |  |
| Self-care | No problems | 270 | 98.96 | 596 | 98.25 | 709 | 97.08 | 710 | 96.02 | 536 | 90.98 | 536 | 91.54 | 345 | 85.96 |
|  | Some problems | 3 | 1.04 | 10 | 1.43 | 21 | 2.92 | 28 | 3.68 | 50 | 8.65 | 48 | 7.80 | 55 | 13.75 |
|  | Unable to | 0 | 0 | 2 | 0.32 | 1 | 0 | 2 | 0.31 | 2 | 0.38 | 3 | 0.67 | 1 | 0.29 |
|  | *Total* | *273* | *100* | *608* | *100* | *731* | *100* | *740* | *100* | *588* | *100* | *587* | *100* | *401* | *100* |
|  | (Missing) | 37 |  | 64 |  | 73 |  | 76 |  | 54 |  | 53 |  | 100 |  |
| Usual activities | No problems | 248 | 91.64 | 545 | 90.46 | 658 | 89.97 | 626 | 84.23 | 450 | 75.89 | 463 | 79.16 | 256 | 63.92 |
|  | Some problems | 23 | 8.36 | 61 | 9.22 | 70 | 9.39 | 111 | 15.31 | 126 | 21.50 | 120 | 19.51 | 134 | 32.67 |
|  | Unable to | 0 | 0 | 3 | 0.32 | 5 | 0.65 | 4 | 0.46 | 15 | 2.62 | 7 | 1.33 | 15 | 3.41 |
|  | *Total* | *271* | *100* | *609* | *100* | *733* | *100* | *741* | *100* | *591* | *100* | *590* | *100* | *405* | *100* |
|  | (Missing) | 39 |  | 63 |  | 71 |  | 75 |  | 51 |  | 50 |  | 96 |  |
| Pain/discomfort | No | 228 | 84.97 | 499 | 83.31 | 546 | 74.43 | 483 | 64.43 | 330 | 55.97 | 310 | 53.24 | 146 | 35.69 |
|  | Some | 40 | 13.21 | 101 | 15.42 | 164 | 22.49 | 237 | 32.52 | 212 | 35.63 | 233 | 39.60 | 225 | 55.52 |
|  | Extreme | 5 | 1.81 | 9 | 1.27 | 21 | 3.07 | 23 | 3.05 | 50 | 8.40 | 44 | 7.16 | 35 | 8.78 |
|  | *Total* | *273* | *100* | *609* | *100* | *731* | *100* | *743* | *100* | *592* | *100* | *587* | *100* | *406* | *100* |
|  | (Missing) | 37 |  | 63 |  | 73 |  | 73 |  | 50 |  | 53 |  | 95 |  |
| Anxiety/depression | No | 206 | 76.04 | 488 | 80.48 | 584 | 79.81 | 578 | 78.80 | 442 | 75.09 | 470 | 80.36 | 309 | 76.72 |
|  | Some | 58 | 21.09 | 103 | 17.14 | 133 | 17.93 | 142 | 18.74 | 131 | 21.72 | 104 | 17.63 | 81 | 20.98 |
|  | Extreme | 8 | 2.86 | 18 | 2.38 | 16 | 2.26 | 19 | 2.46 | 18 | 3.18 | 11 | 2.01 | 10 | 2.30 |
|  | *Total* | *272* | *100* | *609* | *100* | *733* | *100* | *739* | *100* | *591* | *100* | *585* | *100* | *400* | *100* |
|  | (Missing) | 38 |  | 63 |  | 71 |  | 77 |  | 51 |  | 55 |  | 101 |  |

*Percentages may not sum to 100 because of rounding*

**Table S7. HSE2014 Adult males – EQ-5D descriptive dimensions, raw counts, weighted proportions, by age group and dichotomised level of problem**

|  |  | **Age Group** | | | | | | | | | | | | | |
| --- | --- | --- | --- | --- | --- | --- | --- | --- | --- | --- | --- | --- | --- | --- | --- |
| **Total** |  | **18-24y** | | **25-34y** | | **35-44y** | | **45-54y** | | **55-64y** | | **65-74y** | | **>74y** | |
|  |  | *n* | *%* | *n* | *%* | *n* | *%* | *n* | *%* | *n* | *%* | *n* | *%* | *n* | *%* |
| Mobility | No problems | 220 | 94.88 | 364 | 94.69 | 480 | 90.51 | 530 | 87.99 | 393 | 79.92 | 366 | 74.02 | 172 | 55.02 |
|  | Some Problems | 11 | 4.86 | 24 | 5.14 | 51 | 9.49 | 70 | 11.70 | 104 | 19.88 | 129 | 25.74 | 145 | 44.24 |
|  | Confined to bed | 1 | 0.26 | 1 | 0.17 | 0 | 0 | 2 | 0.31 | 1 | 0.20 | 1 | 0.25 | 2 | 0.74 |
|  | *Total* | *232* | *100* | *389* | *100* | *531* | *100* | *602* | *100* | *498* | *100* | *496* | *100* | *319* | *100* |
|  | (Missing) | 32 |  | 67 |  | 75 |  | 63 |  | 69 |  | 54 |  | 59 |  |
| Self-care | No problems | 230 | 99.49 | 381 | 98.80 | 515 | 97.29 | 569 | 95.14 | 459 | 92.37 | 451 | 92.31 | 277 | 87.36 |
|  | Some problems | 1 | 0.26 | 7 | 1.20 | 14 | 2.54 | 29 | 4.55 | 36 | 7.03 | 37 | 7.44 | 38 | 11.52 |
|  | Unable to | 1 | 0.26 | 0 | 0 | 2 | 0.17 | 2 | 0.31 | 3 | 0.60 | 1 | 0.25 | 3 | 1.12 |
|  | *Total* | *232* | *100* | *388* | *100* | *531* | *100* | *600* | *100* | *498* | *100* | *489* | *100* | *318* | *100* |
|  | (Missing) | 32 |  | 68 |  | 75 |  | 65 |  | 69 |  | 61 |  | 60 |  |
| Usual activities | No problems | 214 | 93.32 | 352 | 92.60 | 474 | 89.98 | 522 | 87.64 | 416 | 84.34 | 404 | 82.06 | 218 | 70.19 |
|  | Some problems | 15 | 6.17 | 33 | 7.23 | 50 | 9.17 | 71 | 10.80 | 71 | 13.05 | 78 | 15.48 | 79 | 24.91 |
|  | Unable to | 2 | 0.51 | 1 | 0.17 | 6 | 0.85 | 8 | 1.56 | 10 | 2.61 | 13 | 2.46 | 17 | 4.91 |
|  | *Total* | *231* | *100* | *386* | *100* | *530* | *100* | *601* | *100* | *497* | *100* | *495* | *100* | *314* | *100* |
|  | (Missing) | 33 |  | 70 |  | 76 |  | 64 |  | 70 |  | 55 |  | 64 |  |
| Pain/discomfort | No | 203 | 88.24 | 322 | 84.71 | 391 | 74.41 | 430 | 71.79 | 314 | 63.65 | 288 | 58.09 | 152 | 48.51 |
|  | Some | 29 | 11.76 | 60 | 14.26 | 126 | 23.39 | 152 | 25.24 | 157 | 31.12 | 185 | 37.25 | 149 | 46.64 |
|  | Extreme | 0 | 0 | 5 | 1.03 | 13 | 2.20 | 18 | 2.98 | 26 | 5.22 | 22 | 4.66 | 17 | 4.85 |
|  | *Total* | *232* | *100* | *387* | *100* | *530* | *100* | *600* | *100* | *497* | *100* | *495* | *100* | *318* | *100* |
|  | (Missing) | 32 |  | 69 |  | 76 |  | 65 |  | 70 |  | 55 |  | 60 |  |
| Anxiety/depression | No | 191 | 83.29 | 316 | 81.76 | 446 | 84.04 | 493 | 82.76 | 403 | 82.13 | 433 | 87.53 | 266 | 84.21 |
|  | Some | 36 | 14.91 | 64 | 17.04 | 72 | 13.75 | 93 | 15.05 | 81 | 15.66 | 57 | 11.25 | 48 | 15.04 |
|  | Extreme | 4 | 1.80 | 6 | 1.20 | 11 | 2.21 | 15 | 2.19 | 13 | 2.21 | 6 | 1.22 | 2 | 0.75 |
|  | *Total* | *231* | *100* | *386* | *100* | *529* | *100* | *601* | *100* | *497* | *100* | *496* | *100* | *316* | *100* |
|  | (Missing) | 33 |  | 70 |  | 77 |  | 64 |  | 70 |  | 54 |  | 62 |  |

*Percentages may not sum to 100 because of rounding*

**Table S8. HSE2014 adults ≥45y – EQ-5D descriptive dimensions, weighted counts, proportions and odds, by age-group, sex and dichotomised level of problem**

|  | **Females** | | | | | | | | **Males** | | | | | | | |
| --- | --- | --- | --- | --- | --- | --- | --- | --- | --- | --- | --- | --- | --- | --- | --- | --- |
| **Age Group** | 45-54y | | 55-64y | | 65-74y | | >74y | | 45-54y | | 55-64y | | 65-74y | | >74y | |
| **Mobility** | *n* | *%* | *n* | *%* | *n* | *%* | *n* | *%* | *n* | *%* | *n* | *%* | *n* | *%* | *n* | *%* |
| No problems | 558 | 85.32 | 419 | 78.61 | 327 | 72.83 | 178 | 50.57 | 564 | 87.99 | 398 | 79.92 | 302 | 74.02 | 148 | 55.02 |
| Some problems | 96 | 14.68 | 114 | 21.39 | 122 | 27.17 | 174 | 49.43 | 77 | 12.01 | 100 | 20.08 | 106 | 25.98 | 121 | 44.98 |
| *Total* | *654* | *100* | *533* | *100* | *449* | *100* | *352* | *100* | *641* | *100* | *498* | *100* | *408* | *100* | *269* | *100* |
| OR (95% CI) | *reference* | | **1.65 (1.24 – 2.19)** | | **2.28 (1.74 – 3.01)** | | **6.12 (4.60 – 8.12)** | | *reference* | | **1.97 (1.42 – 2.73)** | | **2.61 (1.90 – 3.59)** | | **6.29 (4.52 – 8.75)** | |
| **Self-care** |  |  |  |  |  |  |  |  |  |  |  |  |  |  |  |  |
| No problems | 627 | 96.02 | 484 | 90.98 | 411 | 91.54 | 300 | 85.96 | 607 | 95.14 | 460 | 92.37 | 372 | 92.31 | 235 | 87.36 |
| Some problems | 26 | 3.98 | 48 | 9.02 | 38 | 8.46 | 49 | 14.04 | 31 | 4.86 | 38 | 7.63 | 31 | 7.69 | 34 | 12.64 |
| *Total* | *653* | *100* | *532* | *100* | *449* | *100* | *349* | *100* | *638* | *100* | *498* | *100* | *403* | *100* | *269* | *100* |
| OR (95% CI) | *reference* | | **2.30 (1.44 – 3.65)** | | **2.25 (1.41 – 3.58)** | | **3.84 (2.42 – 6.10)** | | *reference* | | 1.56 (0.96 – 2.54) | | 1.55 (0.95 – 2.52) | | **2.72 (1.67 – 4.43)** | |
| **Usual activities** |  |  |  |  |  |  |  |  |  |  |  |  |  |  |  |  |
| No problems | 550 | 84.23 | 406 | 75.89 | 357 | 79.16 | 225 | 63.92 | 560 | 87.64 | 420 | 84.34 | 334 | 82.06 | 186 | 70.19 |
| Some problems | 103 | 15.77 | 129 | 24.11 | 94 | 20.84 | 127 | 36.08 | 79 | 12.36 | 78 | 15.66 | 73 | 17.94 | 79 | 29.81 |
| *Total* | *653* | *100* | *535* | *100* | *451* | *100* | *352* | *100* | *639* | *100* | *498* | *100* | *407* | *100* | *265* | *100* |
| OR (95% CI) | *reference* | | **1.71 (1.30 – 2.44)** | | **1.49 (1.13 – 1.97)** | | **3.17 (2.39 – 4.21)** | | *reference* | | 1.29 (0.92 – 1.80) | | **1.49 (1.07 – 2.07)** | | **2.91 (2.08 – 4.08)** | |
| **Pain/Discomfort** |  |  |  |  |  |  |  |  |  |  |  |  |  |  |  |  |
| No problems | 422 | 64.43 | 300 | 55.97 | 238 | 53.24 | 126 | 35.69 | 458 | 71.79 | 317 | 63.65 | 237 | 58.09 | 130 | 48.51 |
| Some problems | 233 | 35.57 | 236 | 44.03 | 209 | 46.76 | 227 | 64.31 | 180 | 28.21 | 181 | 36.35 | 171 | 41.91 | 138 | 51.49 |
| *Total* | *655* | *100* | *536* | *100* | *447* | *100* | *353* | *100* | *638* | *100* | *498* | *100* | *408* | *100* | *268* | *100* |
| OR (95% CI) | *reference* | | **1.47 (1.18 – 1.84)** | | **1.66 (1.33 – 2.07)** | | **3.31 (2.57 – 4.26)** | | *reference* | | **1.47 (1.14 – 1.90)** | | **1.82 (1.41 – 2.34)** | | **2.76 (2.08 – 3.67)** | |
| **Anxiety/Depression** |  |  |  |  |  |  |  |  |  |  |  |  |  |  |  |  |
| No problems | 513 | 78.80 | 401 | 75.09 | 360 | 80.36 | 267 | 76.72 | 528 | 82.76 | 409 | 82.13 | 358 | 87.53 | 224 | 84.21 |
| Some problems | 138 | 21.20 | 133 | 24.91 | 88 | 19.64 | 81 | 23.28 | 110 | 17.24 | 89 | 17.87 | 51 | 12.47 | 42 | 15.79 |
| *Total* | *651* | *100* | *534* | *100* | *448* | *100* | *348* | *100* | *638* | *100* | *498* | *100* | *409* | *100* | *266* | *100* |
| OR (95% CI) | *reference* | | 1.21 (0.94 – 1.56) | | 0.88 (0.67 – 1.15) | | 1.06 (0.79 – 1.42) | | *reference* | | 1.06 (0.78 – 1.45) | | **0.66 (0.47 – 0.93)** | | 0.86 (0.59 – 1.24) | |

**Figure S3. HSE2014 adults ≥45y - EQ5D descriptive dimensions, weighted proportions, by age-group, sex and dichotomised level of problem**

| 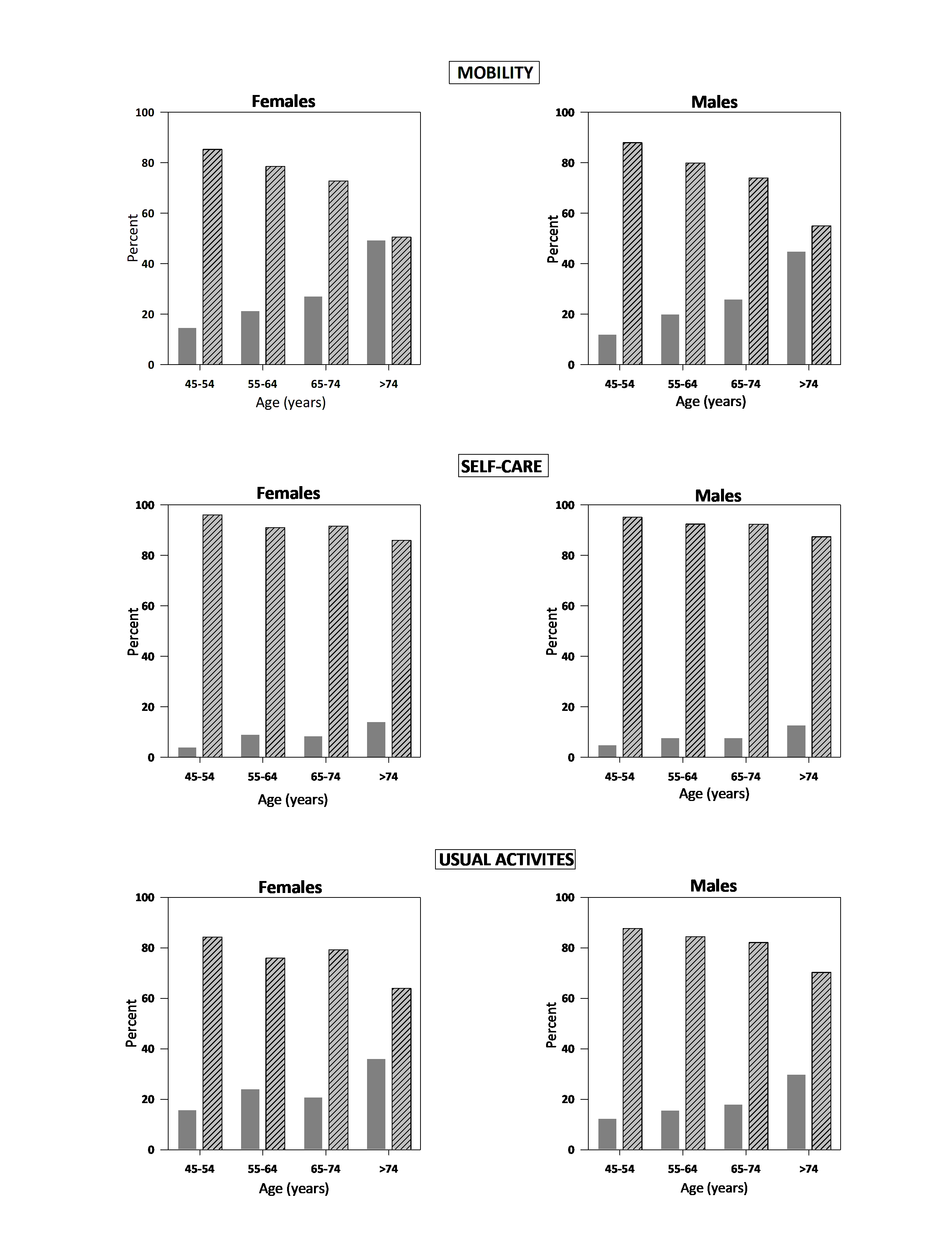 |
| --- |
| 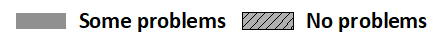 |

**Figure S3 (contd). HSE2014 adults ≥45y - EQ5D descriptive dimensions, weighted proportions, by age-group, sex and dichotomised level of problem**

| 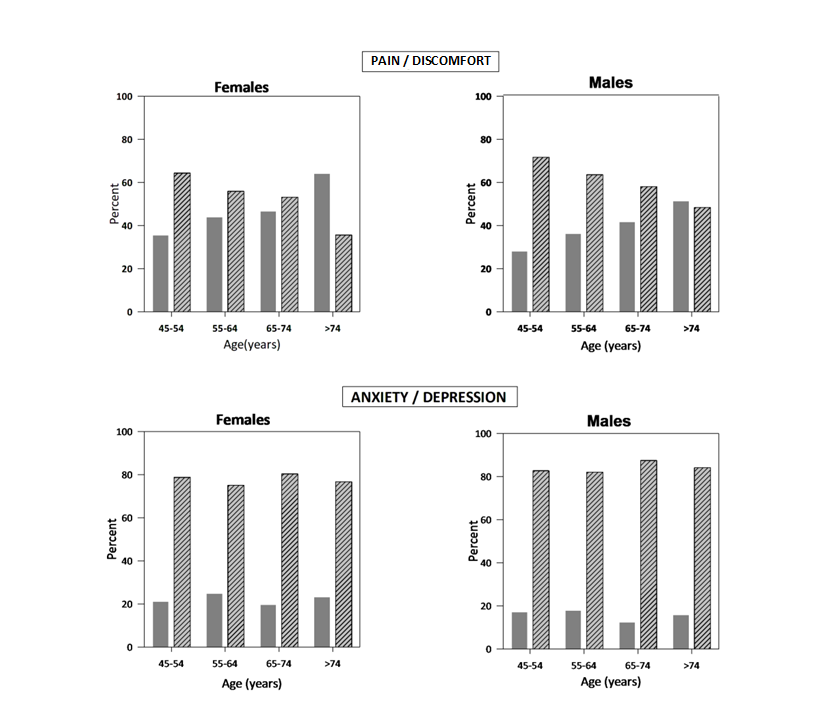 |
| --- |
| 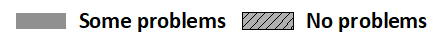 |

**Figure S4. Proportions of participants reporting some problems and no problems, UK Biobank and expected population norm estimates, by EQ-5D descriptive dimension**

| 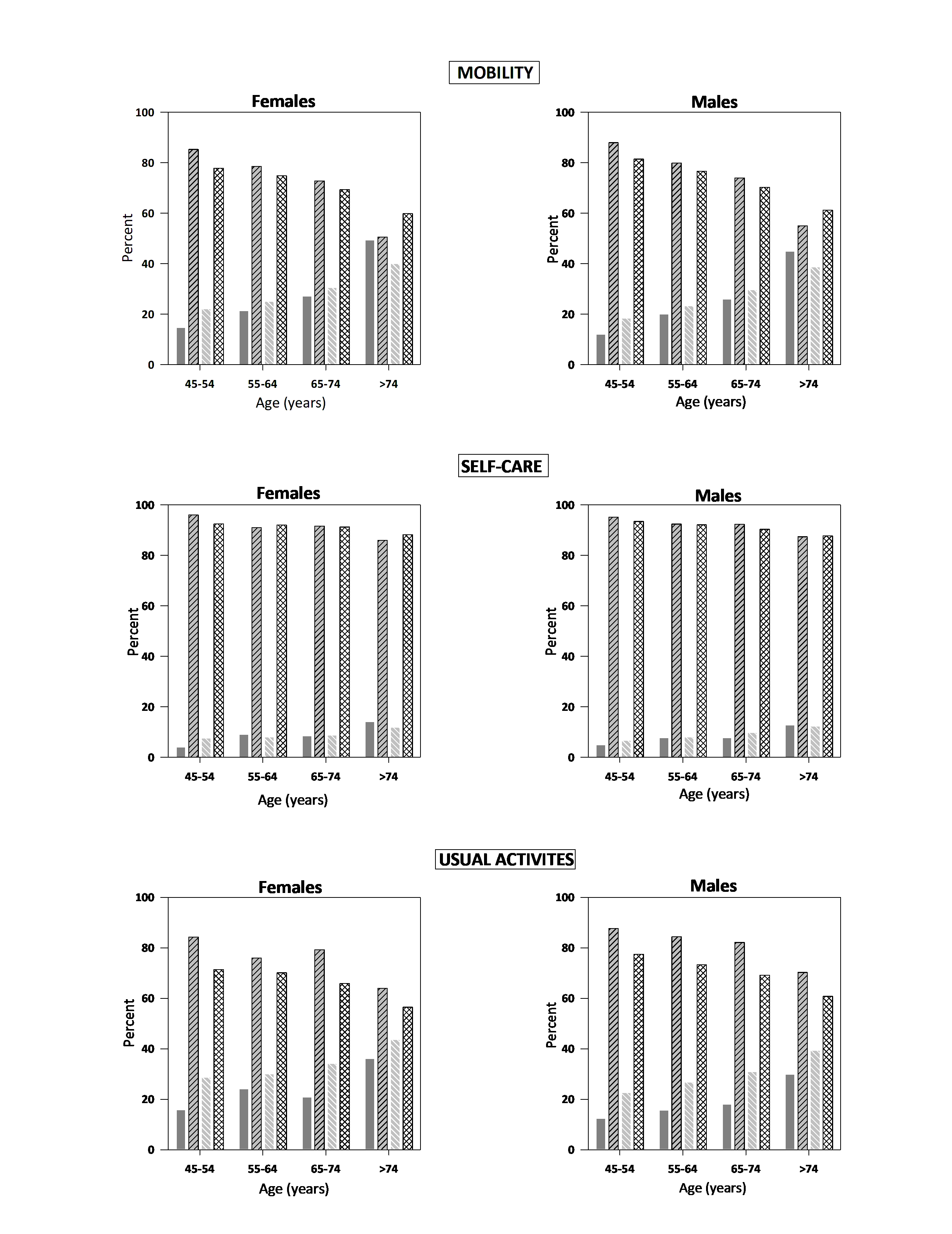 |
| --- |
| 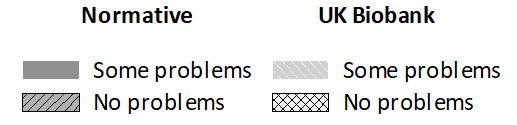 |

**Figure S4 (contd). Proportions of participants reporting some problems and no problems, UK Biobank and expected population norm estimates, by EQ-5D descriptive dimension.**

| 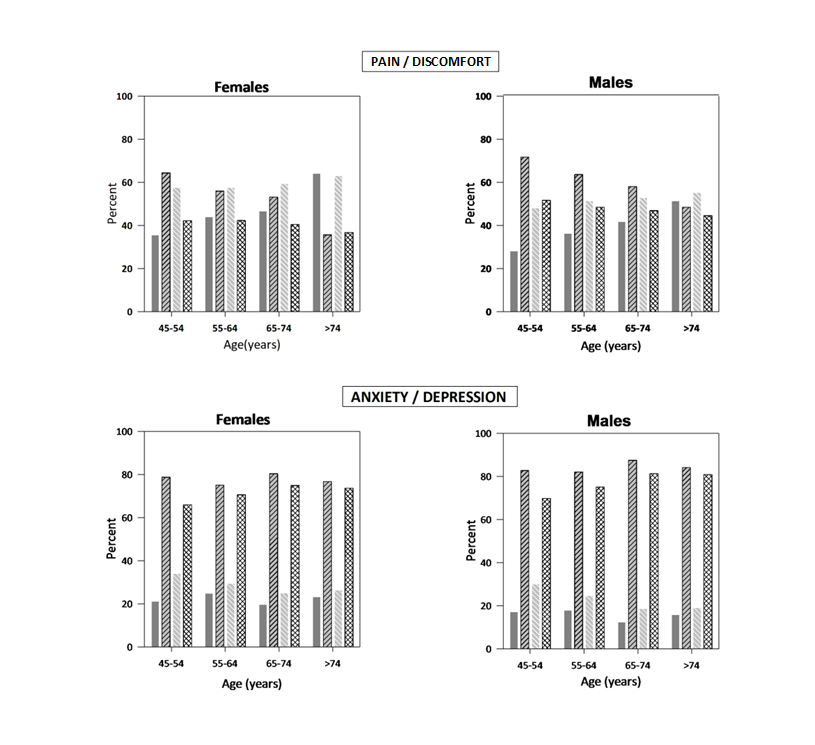 |
| --- |
| 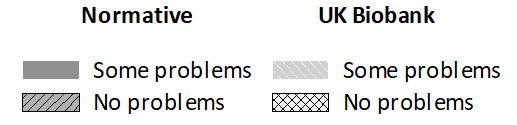 |

**Appendix S4. EQ-5D Health utilities**

**Table S9. UK Biobank - EQ-5D-5L health utility (Devlin 2018 method) by age and sex (n=167,199)**

|  |  | Age Group | | | |
| --- | --- | --- | --- | --- | --- |
|  |  | 45-54y | 55-64y | 65-74y | >74y |
|  |  | *n=13,478* | *n=50,483* | *n=74,850* | *n=28,388* |
| Total  (n=167,199) | Mean | 0.892 | 0.891 | 0.889 | 0.871 |
|  | SD | 0.139 | 0.141 | 0.135 | 0.143 |
|  | 25th percentile | 0.84 | 0.84 | 0.83 | 0.81 |
|  | 50th percentile | 0.94 | 0.94 | 0.94 | 0.92 |
|  | 75th percentile | 1 | 1 | 1 | 1 |
| Females  (n=94,998) | Mean | 0.883 | 0.885 | 0.883 | 0.861 |
|  | SD | 0.145 | 0.142 | 0.138 | 0.147 |
|  | 25th percentile | 0.83 | 0.83 | 0.83 | 0.80 |
|  | 50th percentile | 0.92 | 0.92 | 0.92 | 0.89 |
|  | 75th percentile | 1 | 1 | 1 | 1 |
| Males  (n=72,201) | Mean | 0.904 | 0.899 | 0.898 | 0.880 |
|  | SD | 0.128 | 0.137 | 0.131 | 0.139 |
|  | 25th percentile | 0.86 | 0.86 | 0.84 | 0.83 |
|  | 50th percentile | 0.94 | 0.94 | 0.94 | 0.92 |
|  | 75th percentile | 1 | 1 | 1 | 1 |

**Figure S5. UK Biobank - EQ-5D-5L health utility by age and sex – means and 95% confidence intervals (n=167,199)**


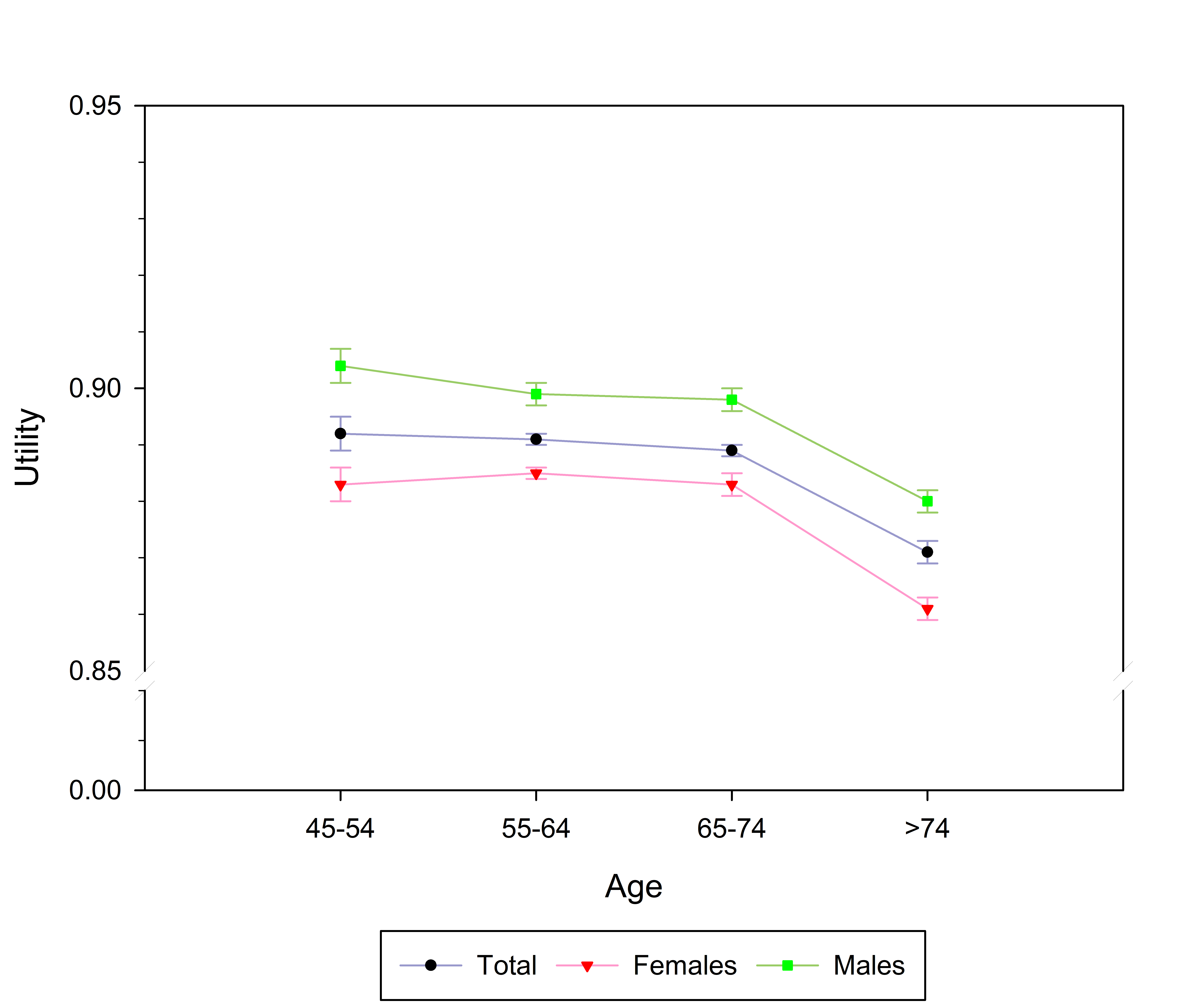


^*^Weights from Devlin NJ, Shah KK, Feng Y, Mulhern B, van Hout B. Valuing health‐related quality of life: An EQ‐5D‐5L value set for England. Health economics. 2018 Jan;27(1):7-22.

**Table S10. UK Biobank - van Hout Crosswalked EQ-5D-3L utilities by age and sex (n=167,199)**

|  |  | Age Group | | | |
| --- | --- | --- | --- | --- | --- |
|  |  | 45-54y | 55-64y | 65-74y | >74y |
|  |  | *n=13,478* | *n=50,483* | *n=74,850* | *n=28,388* |
| Total  (n=167,199) | Mean | 0.834 | 0.833 | 0.831 | 0.809 |
|  | SD | 0.170 | 0.169 | 0.163 | 0.167 |
|  | 25th percentile | 0.75 | 0.74 | 0.74 | 0.72 |
|  | 50th percentile | 0.84 | 0.84 | 0.84 | 0.84 |
|  | 75th percentile | 1 | 1 | 1 | 1 |
| Females  (n=94,998) | Mean | 0.822 | 0.826 | 0.822 | 0.798 |
|  | SD | 0.175 | 0.170 | 0.165 | 0.169 |
|  | 25th percentile | 0.74 | 0.74 | 0.74 | 0.71 |
|  | 50th percentile | 0.84 | 0.84 | 0.84 | 0.80 |
|  | 75th percentile | 1 | 1 | 1 | 1 |
| Males  (n=72,201) | Mean | 0.852 | 0.844 | 0.842 | 0.821 |
|  | SD | 0.162 | 0.167 | 0.159 | 0.164 |
|  | 25th percentile | 0.77 | 0.77 | 0.74 | 0.74 |
|  | 50th percentile | 0.84 | 0.84 | 0.84 | 0.84 |
|  | 75th percentile | 1 | 1 | 1 | 1 |

van Hout B, Janssen MF, et al. Interim scoring for the EQ-5D-5L: Mapping the EQ-5D-5L to EQ-5D-3L value sets. Value in Health 2012 Jul-Aug;15(5):708-15. doi: 10.1016/j.jval.2012.02.008

Dolan P. Modeling valuations for EuroQol health states. Med Care. 1997 Nov;35(11):1095-108. doi: 10.1097/00005650-199711000-00002

**Figure S6. UK Biobank - van Hout crosswalked EQ-5D-3L utilities by age and sex – means and 95% confidence intervals (n=167,199)**


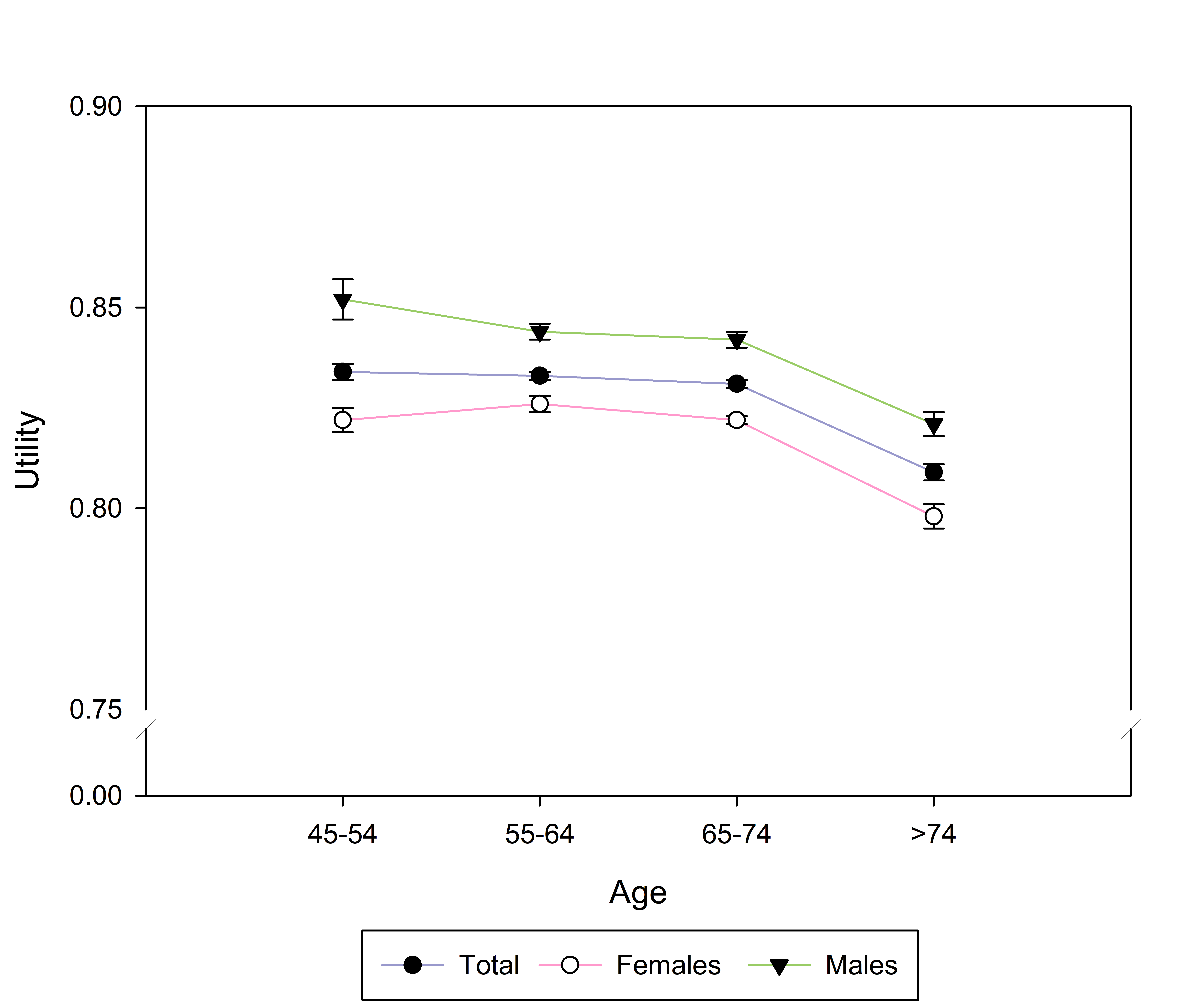


van Hout B, Janssen MF, et al. Interim scoring for the EQ-5D-5L: Mapping the EQ-5D-5L to EQ-5D-3L value sets. Value in Health 2012 Jul-Aug;15(5):708-15. doi: 10.1016/j.jval.2012.02.008; Dolan P. Modeling valuations for EuroQol health states. Med Care. 1997 Nov;35(11):1095-108. doi: 10.1097/00005650-199711000-00002

**Table S11. UK Biobank - DSU method mapped EQ-5D-3L utilities by age and sex (n=167,199)**

|  |  | **Age Group** | | | |
| --- | --- | --- | --- | --- | --- |
|  |  | 45-54y | 55-64y | 65-74y | >74y |
|  |  | *n=13,478* | *n=50,483* | *n=74,850* | *n=28,388* |
| Total  (n=167,199) | Mean | 0.834 | 0.830 | 0.833 | 0.811 |
|  | SD | 0.171 | 0.174 | 0.164 | 0.170 |
|  | 25^th^ percentile | 0.75 | 0.74 | 0.75 | 0.71 |
|  | 50^th^ percentile | 0.86 | 0.86 | 0.87 | 0.84 |
|  | 75^th^ percentile | 0.98 | 0.99 | 0.99 | 0.99 |
| Females  (n=94,998) | Mean | 0.820 | 0.822 | 0.824 | 0.798 |
|  | SD | 0.177 | 0.176 | 0.168 | 0.174 |
|  | 25^th^ percentile | 0.74 | 0.74 | 0.75 | 0.70 |
|  | 50^th^ percentile | 0.86 | 0.86 | 0.87 | 0.80 |
|  | 75^th^ percentile | 0.98 | 0.99 | 0.99 | 0.99 |
| Males  (n=72,201) | Mean | 0.853 | 0.843 | 0.846 | 0.825 |
|  | SD | 0.159 | 0.169 | 0.159 | 0.165 |
|  | 25^th^ percentile | 0.79 | 0.78 | 0.75 | 0.74 |
|  | 50^th^ percentile | 0.86 | 0.86 | 0.87 | 0.87 |
|  | 75^th^ percentile | 0.99 | 0.99 | 0.99 | 0.99 |

**Figure S7. UK Biobank - DSU method mapped EQ-5D-3L utilities by age and sex - – means and 95% confidence intervals (n=167,199)**


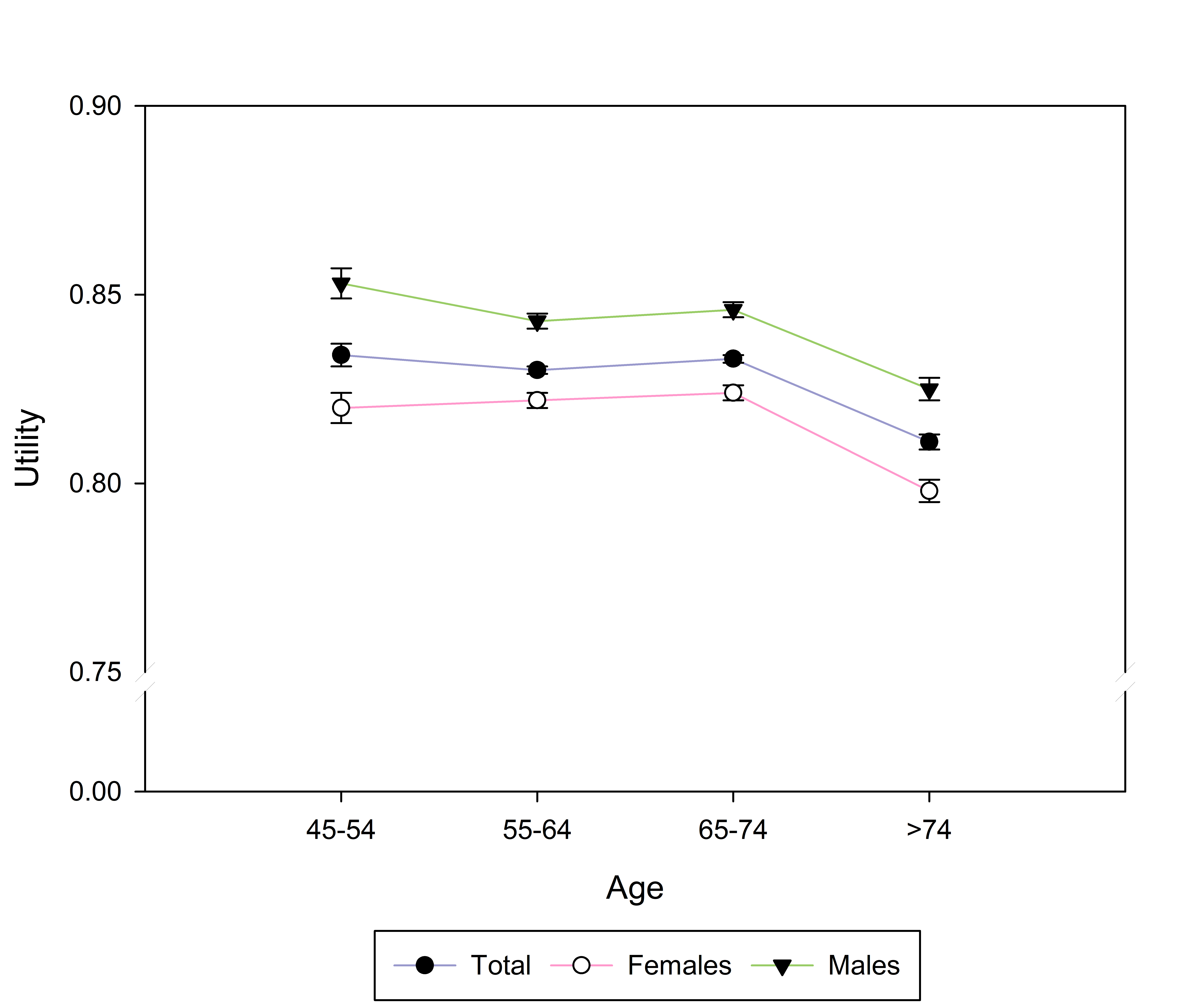


Hernández Alava M, Pudney S, Wailoo A. Estimating the Relationship Between EQ-5D-5L and EQ-5D-3L: Results From an English Population Study [EEPRU Report]. University of Sheffield & University of York; Hernández-Alava M, Pudney S. Econometric modelling of multiple self-reports of health states: The switch from EQ-5D-3L to EQ-5D-5L in evaluating drug therapies for rheumatoid arthritis. J Health Econ. 2017 Sep;55:139-152. doi: 10.1016/j.jhealeco.2017.06.013. Epub 2017 Jul 4

**Table S12. HSE2014 - estimated expected EQ-5D-3L EQ Index values (age 18y-90y) age and sex, using Dolan 1997 value set for the United Kingdom***

| **Females** | | | | | |  | **Males** | | | | | |  | | **EQ Index** | | |
| --- | --- | --- | --- | --- | --- | --- | --- | --- | --- | --- | --- | --- | --- | --- | --- | --- | --- |
| *Age (y)* | *EQ Index* | *Age (y)* | *EQ Index* | *Age* | *EQ Index* |  | *Age (y)* | *EQ Index* | *Age* | *EQ Index* | *Age (y)* | *EQ Index* | |  | *Age (y)* | *Female* | *Male* |
| 18 | 0.915 | 51 | 0.855 | 84 | 0.708 |  | 18 | 0.932 | 51 | 0.874 | 84 | 0.764 | |  | 45-54 | 0.859 | 0.877 |
| 19 | 0.915 | 52 | 0.851 | 85 | 0.702 |  | 19 | 0.931 | 52 | 0.871 | 85 | 0.760 | |  | 55-64 | 0.825 | 0.850 |
| 20 | 0.914 | 53 | 0.848 | 86 | 0.696 |  | 20 | 0.930 | 53 | 0.868 | 86 | 0.755 | |  | 65-74 | 0.783 | 0.818 |
| 21 | 0.913 | 54 | 0.845 | 87 | 0.690 |  | 21 | 0.929 | 54 | 0.866 | 87 | 0.751 | |  | >74 | 0.716 | 0.769 |
| 22 | 0.913 | 55 | 0.841 | 88 | 0.684 |  | 22 | 0.928 | 55 | 0.863 | 88 | 0.747 | |  |  |  |  |
| 23 | 0.912 | 56 | 0.838 | 89 | 0.678 |  | 23 | 0.927 | 56 | 0.860 | 89 | 0.743 | |  |  |  |  |
| 24 | 0.911 | 57 | 0.834 | 90 | 0.672 |  | 24 | 0.925 | 57 | 0.857 | 90 | 0.738 | |  | *Dolan P. *Medical care* 1997: 1095-1108. | | |
| 25 | 0.910 | 58 | 0.831 |  |  |  | 25 | 0.924 | 58 | 0.855 |  |  | |  |  |  |  |
| 26 | 0.909 | 59 | 0.827 |  |  |  | 26 | 0.923 | 59 | 0.852 |  |  | |  |  |  |  |
| 27 | 0.907 | 60 | 0.823 |  |  |  | 27 | 0.921 | 60 | 0.849 |  |  | |  |  |  |  |
| 28 | 0.906 | 61 | 0.819 |  |  |  | 28 | 0.920 | 61 | 0.846 |  |  | |  |  |  |  |
| 29 | 0.905 | 62 | 0.815 |  |  |  | 29 | 0.918 | 62 | 0.843 |  |  | |  |  |  |  |
| 30 | 0.903 | 63 | 0.811 |  |  |  | 30 | 0.917 | 63 | 0.840 |  |  | |  |  |  |  |
| 31 | 0.902 | 64 | 0.807 |  |  |  | 31 | 0.915 | 64 | 0.836 |  |  | |  |  |  |  |
| 32 | 0.900 | 65 | 0.803 |  |  |  | 32 | 0.914 | 65 | 0.833 |  |  | |  |  |  |  |
| 33 | 0.898 | 66 | 0.799 |  |  |  | 33 | 0.912 | 66 | 0.830 |  |  | |  |  |  |  |
| 34 | 0.897 | 67 | 0.794 |  |  |  | 34 | 0.910 | 67 | 0.827 |  |  | |  |  |  |  |
| 35 | 0.895 | 68 | 0.790 |  |  |  | 35 | 0.908 | 68 | 0.823 |  |  | |  |  |  |  |
| 36 | 0.893 | 69 | 0.785 |  |  |  | 36 | 0.907 | 69 | 0.820 |  |  | |  |  |  |  |
| 37 | 0.891 | 70 | 0.781 |  |  |  | 37 | 0.905 | 70 | 0.817 |  |  | |  |  |  |  |
| 38 | 0.889 | 71 | 0.776 |  |  |  | 38 | 0.903 | 71 | 0.813 |  |  | |  |  |  |  |
| 39 | 0.887 | 72 | 0.771 |  |  |  | 39 | 0.901 | 72 | 0.810 |  |  | |  |  |  |  |
| 40 | 0.884 | 73 | 0.766 |  |  |  | 40 | 0.899 | 73 | 0.806 |  |  | |  |  |  |  |
| 41 | 0.882 | 74 | 0.761 |  |  |  | 41 | 0.897 | 74 | 0.802 |  |  | |  |  |  |  |
| 42 | 0.880 | 75 | 0.757 |  |  |  | 42 | 0.895 | 75 | 0.799 |  |  | |  |  |  |  |
| 43 | 0.877 | 76 | 0.751 |  |  |  | 43 | 0.893 | 76 | 0.795 |  |  | |  |  |  |  |
| 44 | 0.875 | 77 | 0.746 |  |  |  | 44 | 0.890 | 77 | 0.791 |  |  | |  |  |  |  |
| 45 | 0.872 | 78 | 0.741 |  |  |  | 45 | 0.888 | 78 | 0.787 |  |  | |  |  |  |  |
| 46 | 0.869 | 79 | 0.736 |  |  |  | 46 | 0.886 | 79 | 0.784 |  |  | |  |  |  |  |
| 47 | 0.867 | 80 | 0.730 |  |  |  | 47 | 0.883 | 80 | 0.780 |  |  | |  |  |  |  |
| 48 | 0.864 | 81 | 0.725 |  |  |  | 48 | 0.881 | 81 | 0.776 |  |  | |  |  |  |  |
| 49 | 0.861 | 82 | 0.719 |  |  |  | 49 | 0.879 | 82 | 0.772 |  |  | |  |  |  |  |
| 50 | 0.858 | 83 | 0.714 |  |  |  | 50 | 0.876 | 83 | 0.768 |  |  | |  |  |  |  |

**Figure S8. HSE2014 adults - EQ-5D-3L index modelled by age and sex using Dolan the 1997 value set for the United Kingdom***

| **Females** | **Males** |
| --- | --- |
| 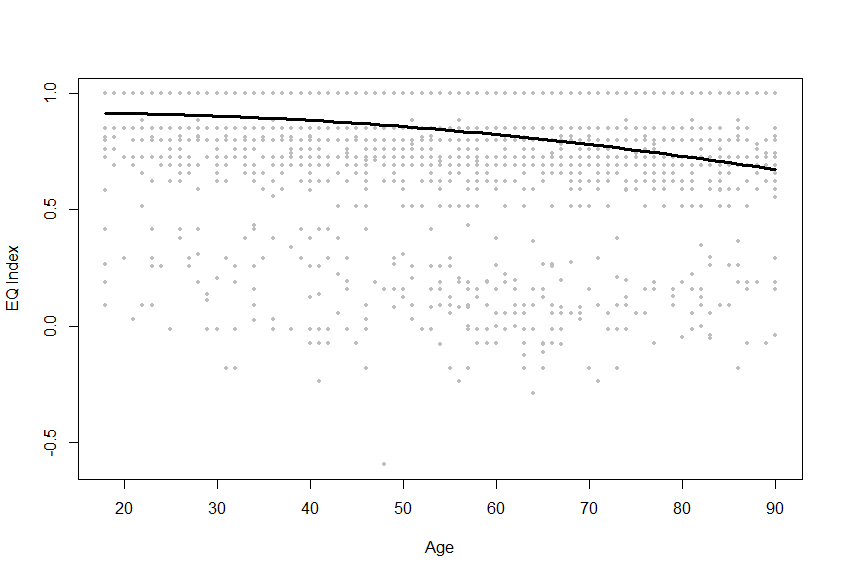 | 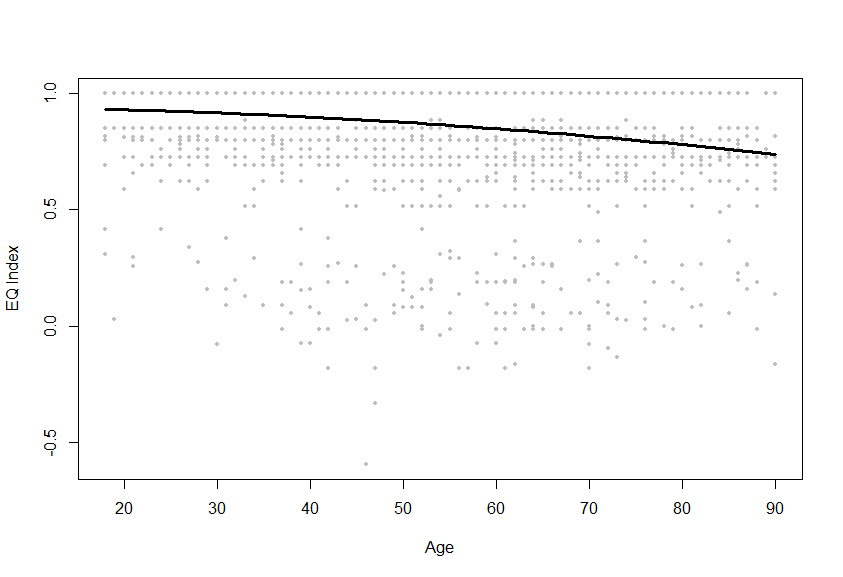 |
| eqindex ~ age + age^2 | |

*Dolan P. *Medical care* 1997: 1095-1108.

**Figure S9. Population utilities from a multi-country analysis (Janssen 2019),* with UK Biobank EoPQ utilities overlaid**

**
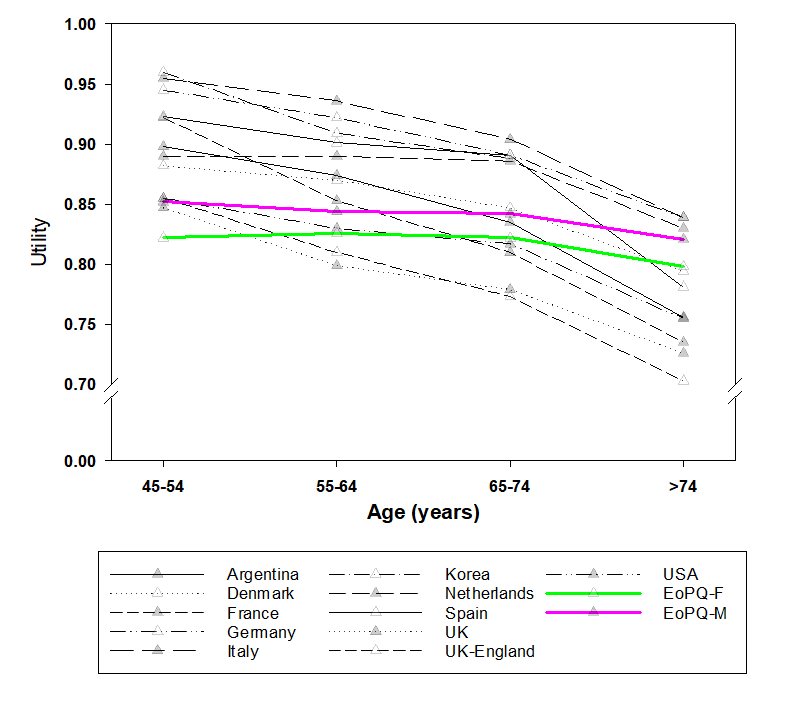
**

Data from: Janssen MF, Szende A, Cabases J, Ramos-Goñi JM, Vilagut G, König HH. Population norms for the EQ-5D-3L: a cross-country analysis of population surveys for 20 countries. The European Journal of Health Economics. 2019 Mar 15;20:205-16.
